# A Review: Methodologies to Promote the Differentiation of Mesenchymal Stem Cells for the Regeneration of Intervertebral Disc Cells Following Intervertebral Disc Degeneration

**DOI:** 10.3390/cells12172161

**Published:** 2023-08-28

**Authors:** Takashi Ohnishi, Kentaro Homan, Akira Fukushima, Daisuke Ukeba, Norimasa Iwasaki, Hideki Sudo

**Affiliations:** 1 Department of Orthopedic Surgery, Faculty of Medicine and Graduate School of Medicine, Hokkaido University, Sapporo 060-8638, Japan; takashi.onishi.ortho@gmail.com (T.O.); kentaro.homan@gmail.com (K.H.); 621akira.fuku@gmail.com (A.F.); niwasaki38@yahoo.co.jp (N.I.); 2 Department of Orthopedic Surgery, Hokkaido University Hospital, Sapporo 060-8648, Japan; daisuke922@nifty.com; 3 Department of Advanced Medicine for Spine and Spinal Cord Disorders, Faculty of Medicine and Graduate School of Medicine, Hokkaido University, Sapporo 060-8638, Japan

**Keywords:** intervertebral disc degeneration, regeneration, mesenchymal stem cell, differentiation

## Abstract

Intervertebral disc (IVD) degeneration (IDD), a highly prevalent pathological condition worldwide, is widely associated with back pain. Treatments available compensate for the impaired function of the degenerated IVD but typically have incomplete resolutions because of their adverse complications. Therefore, fundamental regenerative treatments need exploration. Mesenchymal stem cell (MSC) therapy has been recognized as a mainstream research objective by the World Health Organization and was consequently studied by various research groups. Implanted MSCs exert anti-inflammatory, anti-apoptotic, and anti-pyroptotic effects and promote extracellular component production, as well as differentiation into IVD cells themselves. Hence, the ultimate goal of MSC therapy is to recover IVD cells and consequently regenerate the extracellular matrix of degenerated IVDs. Notably, in addition to MSC implantation, healthy nucleus pulposus (NP) cells (NPCs) have been implanted to regenerate NP, which is currently undergoing clinical trials. NPC-derived exosomes have been investigated for their ability to differentiate MSCs from NPC-like phenotypes. A stable and economical source of IVD cells may include allogeneic MSCs from the cell bank for differentiation into IVD cells. Therefore, multiple alternative therapeutic options should be considered if a refined protocol for the differentiation of MSCs into IVD cells is established. In this study, we comprehensively reviewed the molecules, scaffolds, and environmental factors that facilitate the differentiation of MSCs into IVD cells for regenerative therapies for IDD.

## 1. Introduction

The intervertebral disc (IVD) connects adjacent vertebrae to enable omnidirectional segment motion and absorbs a compressive load or various stains to support the spinal column [1]. Despite the benefits of utilizing this unique tissue, it exhibits complexity and delicacy. IVD degeneration (IDD) occurs secondary to genetic factors [2,3,4,5,6], mechanical overload [7,8,9,10], trauma [11,12,13,14,15], or aging [16,17] and may cause nociceptive pain in the back or neurological deficits, including neuralgia, numbness, and muscular weakness [18]. Currently available treatments include pharmacotherapies using general or neurotropic analgesics, physical therapies, and surgical treatments. These treatments are effective in alleviating symptoms but do not necessarily address the fundamental pathological conditions underlying the disease [19,20]. Furthermore, pharmacotherapies and/or physical therapies can be ineffective when the severity of IDD and accompanying neurological deficits are very advanced. Surgical treatments can often salvage these severe cases, but complications such as relapse of herniation of the nucleus pulposus (NP) [21] or adjacent segment disease after spinal fusion surgeries [22] become problematic in some cases. Accordingly, the ideal treatment for IDD is tissue regeneration, which aims to avoid or minimize the occurrence of sequential detrimental complications. As spine surgeons, we treat patients with IDD every day and encounter such complications, underscoring the importance of developing regenerative treatment for IDD.

Cell therapy has become the mainstream regenerative treatment for degenerated IVD. The World Health Organization has supported regenerative therapies that utilize mesenchymal stem cells (MSCs) and bioscaffolds as primary research objectives [23]. Implanted MSCs have been reported to exhibit anti-inflammatory, anti-apoptotic, and anti-pyroptotic effects, as well as promote extracellular component production and eventual differentiation into IVD cells [24,25,26,27,28,29,30]. While numerous NP cells (NPCs) are required in the environment for the chondrogenic differentiation of MSCs, only a small number of MSCs is sufficient to significantly enhance the proliferation of NPCs, and numerous MSCs are required for the upregulation of aggrecan expression in NPCs [29]. Collectively, the goals of MSC therapy can be recapitulated by the recovery of IVD cells and the consequent regeneration of the extracellular matrix (ECM) of the degenerated IVDs.

Focusing on the harsh environment in the NP, environmental factors, including high osmolarity and low pH, are detrimental for implanted MSCs to survive and express their biological behaviors [31]. The survival of implanted MSCs is an important aspect that may affect treatment outcomes. A previous study reported that human MSCs survived in porcine IVD for at least 6 months, as confirmed by the expression of typical chondrocyte markers [32]. However, it remains unclear whether these anabolic effects persist. Implantation of NPCs is an alternative option. Several clinical trials have previously reported that autologous NPC implantation to degenerate IVDs suppresses the progression of IDD and/or reduces disability levels of patients, in accordance with a reduction in their low back pain [33,34,35]. However, the major obstacle to using autologous NPCs is the difficulty in obtaining donor cells. Unless a plausible reason exists to harvest the NP tissue from an intact IVD, treatment of the degenerated IVD requires sacrificing another intact IVD. For this reason, previous clinical trials that had utilized degenerated IVDs to obtain donor cells and that had selected or activated those cells prior to implantation have only exhibited modest outcomes [33,34,35].

A solution to this complicated problem may be the establishment of a methodology to differentiate allogeneic MSCs in vitro and/or in vivo. MSCs may induce in vitro differentiation into healthy NPCs or annulus fibrosus (AF) cells (AFCs) that have sufficient viability, proliferative potential, and capability for ECM development in the implanted regions. Alternatively, MSC implantation can be performed using strategies aimed at differentiating cells into NPCs or AFCs in situ. This includes implantation in combination with appropriate growth factors, scaffolds, or carriers. The production of decellularized NP or AF matrices is a possible option for in vitro differentiation of MSCs in the production of bioscaffolds.

Based on this background information, we comprehensively reviewed the methodologies underlying the induction of the differentiation of MSCs into IVD cells for regenerative therapies for IDD, including strategies that employ molecules, scaffolds, and environmental factors.

## 2. Efficacies of MSCs on the Pathology of IDD

**Figure 1:**
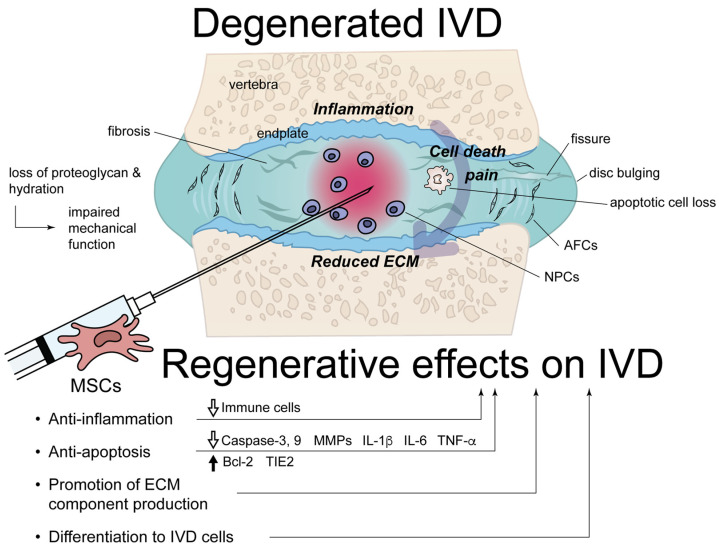
A scheme summarizing the pathological conditions of intervertebral disc (IVD) degeneration and the regenerative effects of mesenchymal stem cells (MSCs). AFC, annulus fibrosus cell; Bcl-2, B-cell lymphoma 2; ECM, extracellular matrix; IL, interleukin; MMP, matrix metalloproteinase; NPC, nucleus pulposus cell; TIE2, tyrosine kinase with Ig and EGF homology domains-2; TNF-α, tumor necrosis factor-α.

In degenerated IVD, pro-inflammatory cytokines are upregulated and trigger regulated cell death and ECM degradation [36,37,38,39]. Cell loss due to regulated cell death and phenotypic changes into hypertrophic chondrocytes leads to ECM fibrosis [19,20,40,41,42,43]. Cell loss in the NP causes a concomitant reduction in proteoglycans with a secondary reduction in hydration and hydrostatic pressure from the NP matrix [43]. Fibrocartilaginous changes in the AF matrix coincide with the loss of fiber tension, buckling, and fissure formation [44]. Cellular senescence also plays an important role in IDD [45,46,47,48]. This process induces the arrest of cellular proliferation, chronic inflammation, and ECM degradation [49]. Many inflammatory factors manifest nociceptive effects, and increased levels of nerve growth factor and brain-derived neurotrophic factor are released in degenerated IVDs [50]. In MSC therapy for IDD, many underlying conditions are affected, including anti-inflammatory, anti-apoptotic, and anti-pyroptotic effects and the promotion of ECM production, in addition to an increase in the number of IVD cells due to MSC differentiation [51,52]. A previous study reported that bone marrow-derived MSCs (BMSCs) promote an increase in endogenous notochordal cells in the NP [30]. Similarly, the conditioned medium of umbilical cord-derived MSCs (UCMSCs) was reported to recover the stemness of NP-MSCs, which is represented by an increase in CD29 and CD105 proteins with an accompanying elevation of *OCT4*, *Nanog*, and *TIE2* mRNAs [53], indicating the effects of extracellular vesicles. This process improves cellular proliferation and chondrogenic differentiation [53]. In addition to the effects of exocytosis/endocytosis, phenomena such as tunneling nanotubes reportedly contribute to subcellular component delivery from MSCs to NPCs, ultimately leading to phenotypic alteration of NPCs [54]. MSCs differentiated into either NPC- or AFC-like cells are considered to regenerate ECMs in the implanted regions. NP and AF have discrete mechanical properties due to endurance in different strains; namely, NP mainly endures compressive loads [1] and AF, tensile, or torsional strains [55]. NPCs produce the ECM enriched by type II collagen, aggrecan, and various small leucine-rich repeat proteoglycan; AFCs are the ones enriched by type I and II collagens, elastin, and fibrillin-1, and all of which characterize the properties of the forming ECM [44]. Although the majority of studies pursue MSC differentiation into NPC-like cells, several studies aimed to differentiate MSCs into AFC-like cells, which are discussed in the later section. The contents of such research studies are summarized in Figure 1.

## 3. Types of MSC Based on Its Source

Representative types of MSC, based on their source, include BMSC [56,57], adipose-derived MSC (ADMSC) [58,59], NP-derived MSC (NPMSC) [60,61], and UCMSC [53,62]. Gou et al. comparatively explained the features of each cell type in their review article [63]. Briefly, harvesting of BMSCs historically has required invasive procedures; however, evidence of improved isolation, culture, and cell therapy using these cells is accumulating [63]. ADMSCs can be obtained abundantly without highly invasive procedures and exhibit low immunogenicity [63]. NPMSCs can be stimulated to proliferate and differentiate in vitro [60] or in situ but may possess suboptimal functions when targeted to degenerated IVDs [63]. Reportedly, UCMSCs possess functions comparable to other types of MSCs [62,64]. Nevertheless, UCMSCs may have a limited chance of application in autologous implantation [63]. However, a recent study successfully accomplished a clinical trial of 1% HA-mounted allogeneic UCMSC implantation to IDD patients with low back pain [65]. Until two years after the injection, the visual analog scales of low back pain significantly reduced, whereas the index of quality of life (Oswestry Disability Index) of the patients significantly improved [65]. Limited studies have compared the functional superiority of different types of MSCs in terms of the differentiation potential into IVD cells. However, a previous study demonstrated that ADMSCs outperformed BMSCs in terms of their ability to differentiate into NPC-like cells in 3D culture, with respect to proliferation, glycosaminoglycan (GAG) and proteoglycan synthesis, and mRNA and protein expression of HIF1-α, GLUT1, SRY-Box Transcription Factor 9 (SOX9), aggrecan, and type II collagen [66]. In addition, Vadala et al. did not detect BMSCs in the NP 3 weeks after direct injection to the IVDs in a rabbit IDD model, and no sign of regeneration, except osteophyte formation, was evident [67]. Interestingly, regarding the differentiation potential of the AF-like phenotype, BMSCs were deemed superior to ADMSCs, with significantly earlier increased expression of *COL I*, *COL II*, and *ACAN* [68].

Advanced technology has enabled the sorting of highly proliferative BMSCs, such as rapidly expanding clones (RECs), based on the cell surface markers CD271 and CD90 [57]. These cells exhibit less variability and more uniform phenotype/function compared to commercial human BMSCs, thus potentially allowing improved quality for cell therapy [57].

## 4. Factors to Induce the Differentiation of MSCs into IVD Cells

**Figure 2:**
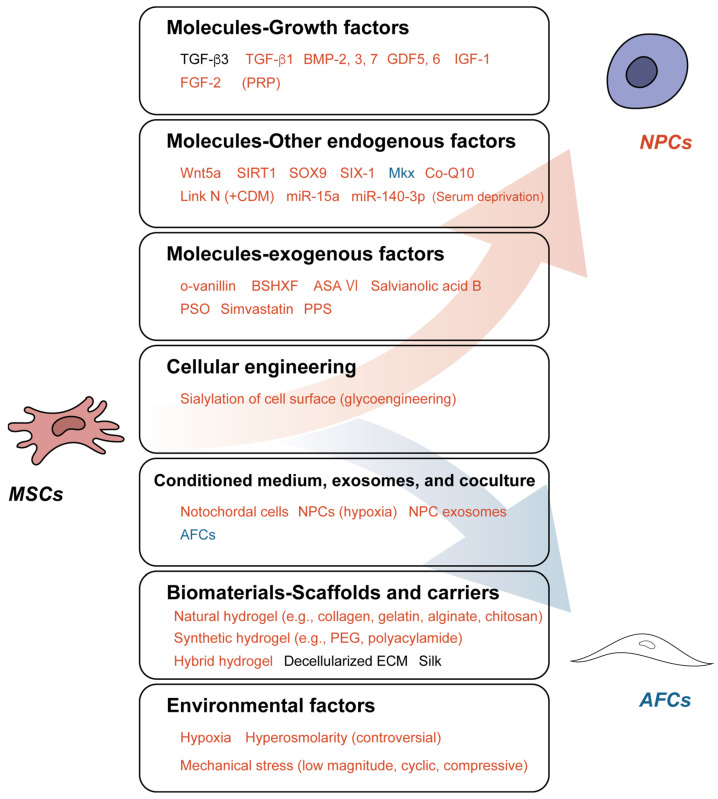
Various strategies to differentiate mesenchymal stem cells (MSCs) into nucleus pulposus cells (NPCs) and annulus fibrosus cells (AFCs). Factors in red induce MSC differentiation toward NPC/chondrogenic phenotype; factors in blue, AFC phenotype; factors in black, either NPC/AFC phenotype. ASA VI, Asperosaponin VI; BMP, bone morphogenic protein; BSHXF, BuShenHuoXueFang; CDM, chondrogenic differentiation medium; Co-Q10, Coenzyme Q10; ECM, extracellular matrix; FGF-2, fibroblast growth factor-2; GDF, growth differentiation factor; IGF-1, insulin-like growth factor-1; miR, microRNA; Mkx, Mohawk; O-vanillin, Ortho-vanillin; PEG, polyethylene glycol; PPS, pentosan polysulfate; PRP, platelet-rich plasma; PSO, psoralidin; SIRT1, silent mating type information regulator 2 homolog 1; SIX-1, sine oculis homeobox homolog-1; SOX9, SRY-Box Transcription Factor 9; TGF, transforming growth factor; Wnt5a, Wingless signaling transduction 5a.

To elicit the potential for MSCs to differentiate into IVD cells of interest, multiple factors can be applied, including molecules, scaffolds, and environmental factors. This section introduces each factor and organizes previously investigated findings. Figure 2 summarizes the content of this whole section.

### 4.1. Molecules—Growth Factors

Transforming growth factor (TGF)-β3 is a growth factor contained in the classical chondrogenic differentiation medium with L-proline, pyruvate, insulin–transferrin–selenium solution, and L-ascorbic acid 2 phosphate [64,69,70]. TGF-β3 is studied to differentiate MSCs into both NPCs and AFCs. A spheroid culture of BMSCs with TGF-β3, dexamethasone, and ascorbate led to the positive expression of type II collagen and *ACAN*, *DCN*, *FMOD*, and *COMP*, which was similar to levels expressed in NP tissue [71]. This culture was then used to assist the co-culturing of ADMSCs and NPCs in differentiating ADMSCs toward the NPC phenotype [72]. Other growth factors, such as bone morphogenic protein (BMP)-2 and insulin-like growth factor (IGF)-1, have been used in combination to synergistically support the effects of TGF-β3 [73,74]. The combination of TGF-β3 and BMP-2 induced chondrogenic differentiation in alginate bead-encapsulated BMSCs that had been cultured in a serum-free medium with a marked upregulation of *ACAN* and *COL2A1* [73]. IGF-1 was also synergistically affected along with TGF-β3 and enabled the differentiation of NPMSCs into NPCs, partially via the activation of the MAPK/ERK signaling pathway [74]. Gruber et al. aimed to induce the differentiation of MSCs toward AFC-like phenotypes. In combination with a 3D culture, TGF-β3 supplementation resulted in the chondrogenic differentiation of ADMSCs [75,76].

Another isoform of TGF-β, TGF-β1, is reported to contribute to MSC differentiation toward an NP/chondrogenic phenotype [59,77]. BMSCs can differentiate into NPC-like phenotypes in 3D nanofibrous poly-L-lactide scaffolds under 2% O2 hypoxia in the presence of TGF-β1 [77]. Risbud et al. also reported that hypoxia augmented the effect of TGF-β1 [78]. Hypoxia played a role in maintaining the expression of endoglin, which is the TGF-β receptor in rat MSCs that had been cultured in 3D alginate hydrogels [78]. Furthermore, TGF-β1 treatment upregulates MAPK activity, specifically ERK1/2, *SOX9*, *ACAN*, and *COL2* gene expression [78]. The synergistic effects of TGF-β1 with growth differentiation factor (GDF) 5 promoted human ADMSCs to differentiate into an NP-like phenotype, as shown by gene expression pattern and ECM production, which were determined to be via the Smad 2/3 signaling pathway [59,79]. Meanwhile, Notch 1 knockdown supported the effect of TGF-β1 regarding the enabling of the chondrogenic differentiation of MSCs [80]. Chondrogenic differentiation was also observed in TGF-β1-transfected BMSCs that had been cultured in calcium alginate gel microspheres under simulated microgravity conditions using a rotary cell culture system [81]. Some studies previously reported the efficacy of platelet-rich plasma (PRP) [82] in inducing chondrogenic differentiation or differentiating MSCs into an NP-like phenotype [83,84]. The effects of PRP are considered dependent on growth factors, including TGF-α and β, platelet-derived growth factors [85], and vascular endothelial growth factors. However, aggrecan, collagen types I and II, and SOX9 were less expressed in terms of gene and protein levels when MSCs were cultured with PRP compared to simple TGF-β1, indicating that PRP may not be recommended for MSC differentiation [84].

Other members of the TGF-β superfamily include BMPs [86], and several isoforms have been reportedly involved in the differentiation of MSCs into an NP-like phenotype or chondrogenic differentiation. BMP-2 was utilized with simulated periodic mechanical stress and a chondrogenic differentiation medium and exerted a positive effect on NPMSC differentiation toward NPC [70]. Utilizing BMP-2 in combination with TGF-β3 was found to adequately enhance the chondrogenic differentiation of BMSCs that had been cultivated in alginate beads in a serum-free medium [73]. BMP-2-transduced BMSCs cultured in PRP gels also promoted the chondrogenic differentiation of BMSCs [87]. Another isoform, BMP-3 supplementation after pretreatment with IL-1β, was proven to enhance human MSC proliferation and chondrogenic differentiation [88]. BMP-7 was overexpressed in BMSCs via vector transduction, which induced NP-like differentiation through the Smad pathway [89]. In a comparative study of BMP-2 and BMP-7, BMP-2 was suggested to induce osteogenic differentiation, whereas BMP-7 induced chondrogenic differentiation of ADMSCs [90]. *RUNX2* and *SPP1* were found to be upregulated by BMP-2 but not BMP-7, and *ACAN* was found to be upregulated only by BMP-7 treatment [90], suggesting that BMP-2 induces osteogenic rather than chondrogenic differentiation.

GDF5 and 6 are expected to replace TGF-β regarding the efficacy of differentiating MSCs into an NPC-like phenotype. GDF5 transfection or supplementation exhibited an effect on NPMSC [91] and BMSC [79,92,93] differentiation into NP-like cells. In another study, GDF5 was electroporated into BMSC cultured in 1.2% alginate beads, which successfully exhibited chondrogenic differentiation [94]. Human recombinant GDF6 has been reported to differentiate both BMSCs and ADMSCs into an NP-like phenotype [95]. Notably, a previous study showed that GDF6 outperformed GDF5 or TGF-β3 in terms of the differentiation potential of both BMSCs and ADMSCs into an NP-like phenotype [96]. Other studies have investigated the effect of GDF6 on MSCs embedded in carriers, such as poly(DL-lactic acid-co-glycolic acid), (PLGA)-polyethylene glycol-PLGA microparticles, or a combination of poly(N-isopropylacrylamide-graft-chondroitin sulfate) hydrogel and alginate microparticles, and revealed the role of BMSC and ADMSC differentiation toward an NP-like phenotype [97,98].

Several other growth factors have also been reported to play a role in MSC differentiation. Insulin-like growth factor (IGF)-1 supplemented the culture of human MSCs to render NPC-like differentiation [85,99]. Fibroblast growth factor (FGF)-2 is a potent mitogenic factor and, when cultivated in alginate, is reported to play a role in maintaining the NPC phenotype via a TGF-β1 response [100]. It also induces MSC differentiation into either the NPC-like or chondrogenic phenotypes [85,101]. However, the opposite effect of FGF-2 has also been reported; namely, novel NP markers decreased in FGF-2-supplemented cultures. This suggests that FGF-2 exhibits an overall controversial role in terms of MSC differentiation into an NPC-like phenotype [102]. Table 1 summarizes the content of this section.

### 4.2. Molecules—Other Endogenous Factors

Wnt, a cysteine-rich endogenous glycoprotein, is encoded by 19 genes of the human genome [103,104]. Wnt signaling is recognized as an important player during IVD development and has pivotal effects depending on canonical or noncanonical signaling as well as cell- and tissue-specific signaling [105]. Focusing on the chondrogenic differentiation of MSCs, the effect of Wnt3a is controversial [105]. Although various growth factors, such as TGF-β1, 3, BMP-2, and FGF-2, are used in combination, some studies have shown positivity [106,107] and others have shown negativity [108,109,110,111]. In contrast, Wnt5a positively affected the chondrogenic differentiation of MSCs [105,106,111,112,113]. Treatment with lithium chloride promotes the differentiation of ADMSCs toward the NPC-like phenotype, presumably due to augmentation of the glycogen synthase kinase 3β-dependent β-catenin/Wnt pathway [114].

Silent mating type information regulator 2 homolog 1 (SIRT1) is an NAD^+^-dependent deacetylase that deacetylates histones and other molecules [115,116]. It is involved in a broad range of cellular processes such as apoptosis, autophagy, and inflammation; however, its role in preventing cell senescence and prolonging the lifespan of an organism is especially underscored [115,116,117]. SIRT1 promotes the chondrogenic differentiation of NPMSCs by downregulating the monocyte chemoattractant protein 1 and chemokine receptor 2 axis [117].

SOX9 regulates MSC differentiation into chondrocyte-like cells [118]. The conditional knockout of *SOX9* in *ACAN*-expressing cells resulted in the progressive degeneration of all compartments of the IVD, including the cartilaginous endplate [119]. When *SOX9* was transfected into BMSCs cultivated in porous biodegradable three-dimensional (3D) poly-L-lactic acid scaffolds, the cells differentiated into an NPC-like phenotype, generating type II collagen and aggrecan [118]. Sine oculis homeobox homolog 1 (SIX-1) is a transcription factor that is expressed during the development of limb tendons [120,121]. In a study in which *SOX9* and *SIX1* were overexpressed in UCMSCs, the cells ultimately exhibited chondrogenic differentiation with an enhancement in the expression of *TGFB1*, *BMP*, *SOX9*, *SIX1*, and *ACAN* [122].

Mohawk (Mkx) is a homeobox protein that is a key transcription factor and regulator of AF development, maintenance, and regeneration, and is mainly expressed in the outer AF [123]. Accordingly, *Mkx* was overexpressed in MSCs to ultimately determine whether differentiation into AFC-like cells occurred. The results indicated that MSCs were differentiated toward the AFC-like phenotype, thereby resulting in enhanced type I collagen and decorin mRNA and protein levels, possibly via the TGFβ/Smad signaling pathway rather than the BMP/Smad signaling pathway [123].

Coenzyme Q10 (Co-Q10) is an endogenous lipophilic molecule, and also known as ubiquinone (2,3-dimethoxy-5-methyl-6-polyprenyl-1,4-benzoquinone) [124]. It is found in the phospholipid bilayer of cellular membranes and is especially localized in the mitochondrial inner membrane, where it serves as a component of the mitochondrial electron transport chain [124,125]. The main effect of Co-Q10 is the inhibition of mitochondrial ROS generation and the subsequent prevention of cellular senescence, which is also applicable to stem cells [126]. To resolve the challenges of utilization due to the hydrophobic nature of Co-Q10, it was coated with a phospholipid molecule, namely lecithin, to render it hydrophilic and treat BMSCs. The results showed that Co-Q10 protected BMSCs from oxidative stress and promoted their differentiation toward an NP-like phenotype [124].

Link N is the N-terminal peptide of the link protein that stabilizes the interaction between aggrecan and hyaluronan [127]. It is generated during proteolytic degeneration in vivo and has an agonistic effect on collagen synthesis in NP and AF pellet cultures [127,128]. Although Link N alone did not induce MSC chondrogenesis, it was inductive when applied together with a chondrogenic differentiation medium, resulting in increased GAG secretion, the upregulation of *ACAN*, *COL2A1*, and *SOX9* expression, and the downregulation of *COL10A1* and *BGLAP* expression [129].

MicroRNAs (miRNAs) are small non-coding RNAs comprising 15–30 nucleotides that function as post-transcriptional inhibitors of gene expression. Numerous studies have reported that they play an important role in the process of IDD [43,130]. Most miRNAs are involved in the promotion or suppression of regulated cell death in IVD cells [43]; however, a few contribute directly to the differentiation of MSCs. miR-15a is also known to modulate the expression of genes involved in cellular proliferation and apoptosis [43,130]. Moreover, this miRNA has been studied for its role in the chondrogenic differentiation of NPMSCs. It was transfected into NPC-derived exosomes and used to treat NPMSCs, resulting in increased aggrecan and type II collagen mRNA and protein levels, whereas mRNA and protein levels of ADAMTS4/5 and MMP-3/-13 decreased [131]. Further studies revealed that this effect is mediated through the PI3K/Akt and Wnt3a/β-catenin axes [131]. Another miRNA, termed miR-140-3p, is also downregulated in degenerative IVDs [132,133]. Based on this study, the effect of miR-140-3p overexpression on the progression of IDD was assessed. The overexpression of miR-140-3p alleviates IDD by targeting Kruppel-like factor 5 (KLF5), which interferes with the migration and differentiation of MSCs [133]. NPMSCs from degenerated IVDs were facilitated to differentiate into NPCs through the inhibition of the KLF5/N-cadherin/mouse double minute 2/Slug axis [133].

The role of serum supplementation in cell culture remains largely unknown, although numerous humoral factors are thought to be involved [134]. Interestingly, serum deprivation seemed to be optimal for inducing chondrogenic differentiation of MSCs. The conditions for culturing ADMSCs with or without fetal bovine serum (FBS) were evaluated [135]. Although FBS-free conditions allow ADMSCs to survive, proliferate, and undergo adipogenic, osteogenic, and chondrogenic differentiation, ADMSCs cultured without FBS have enhanced potential for chondrogenic differentiation [135]. Table 2 summarizes the content of this section.

### 4.3. Molecules—Exogenous Factors

Ortho-vanillin (o-vanillin) is a natural compound that inhibits toll-like receptors, thereby preventing inflammation [136]. O-vanillin exhibits senolytic properties and augments the proliferation of non-senescent cells, which consequently increases ECM synthesis in degenerated IVDs [137]. In another study, the conditioned medium of o-vanillin-treated human IVD cells (NPCs and inner AFCs) induced the chondrogenic differentiation of human MSCs, as shown by the elevation of *FOXF1*, *PAX1*, *TIE2*, *SOX9*, *HIF1A*, and *ACAN* gene expression compared to the control [138].

BuShenHuoXueFang (BSHXF) is a Chinese herbal formula that has been reported to improve the environment of degenerated IVD, enhance NPC proliferation, and delay IDD progression [139]. Therefore, the role of BSHXF-medicated serum in MSC differentiation was examined, and ADMSCs exhibited differentiation toward an NPC-like phenotype [140].

Asperosaponin VI (ASA VI) is an herbal Chinese traditional medicine with a long history of safe use in strengthening tendons and bones [141]. The ERK1/2 and Smad2/3 signaling pathways regulate the differentiation of NPMSCs into NP-like cells [74], and ASA VI modulates these pathways [142]. Hence, ASA VI was assessed for its effects on human MSCs, and it was confirmed that MSCs differentiate into NP-like cells [141].

Salvianolic acid B is a compound of *Radix Salvia miltiorrhiza* extracted from the roots of *S. miltiorrhiza* and is similar to “Danshen”, which is another traditional Chinese medicine [143]. It is known as a reactive oxygen species scavenger and an inhibitor of inflammation and metalloproteinase expression in aortic smooth muscle cells [143]; therefore, it has been used to treat cardiovascular diseases in China [144]. Based on previous studies, salvianolic acid B was assessed whether it promotes MSC differentiation in the context of NP regeneration. Salvianolic acid B treatment increased the type II collagen, proteoglycan, TGF-β1, and water content of MSC-implanted IVDs compared to the control, suggesting its ability to enhance the chondrogenic differentiation of MSCs in vivo [145].

Psoralidin (PSO) is the main bioactive compound in the traditional medicine, *Cullen corylifolium* (L.) Medik [146]. PSO has been identified in the seeds of medicinal plants. Cullen corylifolium grows in Asia, India, and Europe [147]. PSO has various anti-inflammatory, antibacterial, antioxidant, antipsoriatic, antidepressant, estrogenic-like, and antitumor properties, and may also stimulate osteoblast proliferation [146]. Considering the results of previous studies, PSO was investigated for its effect on ADMSC differentiation, and differentiation toward an NPC-like phenotype was confirmed [148].

Simvastatin is an approved medicine for hyperlipidemia; however, previous studies have elucidated its effect on inhibiting NPC apoptosis and preventing IDD [149,150]. Furthermore, simvastatin was reported to drive osteogenic differentiation and the migration of BMSCs [151,152]. Based on these results, the effect of simvastatin was explored on the differentiation potential of NPMSCs. This research demonstrated that NPMSCs successfully differentiated into NPC-like phenotypes following treatment with simvastatin [61].

Pentosan polysulfate (PPS) is a semi-synthetic sulfated xylan isolated from beech trees that acts similarly to heparan sulfate in vivo [153]. It has been used to treat interstitial cystitis [154] and is an anti-arthritic drug for coxalgia [153]. The mechanism of this anti-inflammatory effect is considered to be the inhibition of complement activation via C-reactive proteins and the aggregation of IgG [155]. In addition, PPS regulates coagulation [156], fibrinolysis [157], thrombocytopenia [158], the synthesis of hyaluronan [159], the inhibition of nerve growth factor production in osteocytes [160], and the stimulation of proteoglycan synthesis in chondrocytes [161,162]. This multifactorial mucopolysaccharide derivative also has the potential to induce the chondrogenic differentiation of BMSCs. After treating BMSCs with PPS, PPS was successfully internalized by BMSCs and consequently augmented both cell proliferation and proteoglycan synthesis [163,164]. The application of PPS-treated BMSCs to degenerated IVDs with a collagen sponge inhibited the IDD processes in an ovine model of lumbar microdiscectomy [164]. Table 3 summarizes the content of this section.

### 4.4. Cellular Engineering

The surface of ADMSCs was functionalized with unnatural sialic acid via metabolic glycoengineering, and it was examined whether this cellular engineering improved the specificity of ADMSC differentiation toward the NPC-like phenotype. The results showed elevated NPC markers, namely *SOX9*, *COL2*, *KRT19*, and *CD24* expression [165]. Consistently, the implantation of glycoengineered ADSCs improved the height, biomechanical properties, and histological findings of the treated IVDs [165].

### 4.5. Conditioned Mediums, Exosomes, and Co-Cultures

Interactions between different cell types cause reciprocal phenotypic changes. Humoral factors secreted from cells, either directly or indirectly encapsulated in extracellular vesicles, such as exosomes, are a form of cellular communication [166,167]. Another method of cellular communication involves tunneling nanotubes, in which the transfer of subcellular materials occurs [54]. In this section, we introduce methodologies for the chondrogenic differentiation of MSCs using a conditioned medium, exosome, and co-culture with IVD cells.

The conditioned medium of notochordal cells (NCCM) exhibited a strong effect on the chondrogenic differentiation of BMSCs. NCCM resulted in significantly higher GAG accumulation than either the control medium or the chondrogenic differentiation medium [168]. While the NPC-conditioned medium (NPC-CM) does not exhibit a consistent trend of MSC differentiation under normoxia, NPC-CM in combination with hypoxia (2% O_2_) consistently revealed an upregulation of *ACAN*, *TBXT*, *COL2*, *KRT8*, *KRT19*, and *SHH* in BMSCs [169].

NPC exosomes may play a factor in the effect of NPC-CM and actually promote the differentiation of BMSCs into an NPC-like phenotype, as demonstrated by the upregulation of *ACAN*, *SOX9*, *COL2A1*, *HIF1A*, *CA12*, and *KRT19* expression [170]. Moreover, the upregulation of aggrecan, type II collagen, Sox-9, CA12, and KRT19 protein levels has also been established [170]. However, the direct treatment of BMSCs with NPC exosomes was more effective in differentiating BMSCs into NPC-like cells compared to the trans-well co-culture of BMSCs with NPCs [171]. The effect of the trans-well co-culture was demonstrated to occur through NPC exosomes by confirming the role of Rab27a, an important protein in the process of exosome secretion [171]. However, the discrepancy in the potential between co-cultures and exosomes was possibly due to the lower concentration of exosomes released by NPCs in the co-culture method [171]. The effect of NPC exosomes on BMSC differentiation was further shown to be mediated by the Notch 1 pathway [171]. Although the Hypoxia/HIF-1α-Notch signaling pathway plays an important role in cell proliferation and the self-renewal of NPCs [172,173,174,175,176], the Notch signaling pathway exhibited a negative role in the expression of ECM component genes, including *COL2*, *ACAN*, and *SOX9* [171]. Similar results were confirmed by Notch1 knockdown in combination with TGF-β1 treatment, resulting in the upregulation of proteoglycan and type II collagen expression in rabbit MSCs [80].

The co-culture of MSCs with NPCs is often used to differentiate MSCs into NPC-like cells. Both direct co-culture [56,57,177,178,179,180] and trans-well co-culture [72,181,182,183] successfully induced the chondrogenic differentiation of MSCs. Wharton’s jelly is another source of MSCs [184]. Both the direct and trans-well co-cultures of Wharton’s jelly cells with NPCs induced the differentiation of MSCs to NP-like cells, but the gene expression levels of *ACAN*, *COL2*, and *SOX9* were higher in the direct co-culture group [184]. The co-culture of these cells in special settings has also been investigated. Synergistic effects on chondrogenic differentiation were observed in the dynamic compression and co-culture of ADMSCs with NPCs at a 12 h intermittent dynamic hydrostatic pressure of 17 kPa [185]. In the bilaminar cell pellet, where a sphere of MSCs forms the core and shell, NPCs increased MSC proliferation and chondrogenic differentiation compared to single cell-type pellets or randomly mixed co-culture pellets [186]. The co-culture of MSCs with AFCs has also been confirmed to induce MSC differentiation toward AFC-like cells. Similarly, both direct co-culture [76] and trans-well co-culture [75] successfully induced MSC differentiation into AFC-like cells. A comparison of differentiation efficiency toward AFC-like phenotypes revealed the superiority of BMSCs over ADMSCs when direct co-culture was performed with AFCs [68]. The co-culture of rat BMSCs with IVD tissue, including the inner NP, outer AF, and part of the endplate, promoted the chondrogenic differentiation of BMSCs, as evidenced by the expression of type II collagen, aggrecan, Sox-9 mRNA, and protein levels [187]. Table 4 summarizes the content of this section.

### 4.6. Biomaterials—Scaffolds and Carriers

Numerous studies have reported the efficacy of biomaterials that function as scaffolds or carriers for implanted MSCs to undergo differentiation and/or proliferation. Hydrogels are the most widely studied materials for this purpose. Cell and hydrogel interactions influence cell reactions such as differentiation, proliferation, and migration [188]. The compositions of hydrogels are more than 90% water [188] with the following diverse additives: natural materials, including collagen [189,190], gelatin [191,192], hyaluronic acid (HA) [193], alginate [194], fibrin [195], chitosan [196,197,198], agarose [199], polypeptide [32,200], PRP [193], PRP/HA/batroxobin (anticoagulant and gelling agent reactive to PRP) [201], and multiple materials combined [202,203], and synthetic materials, including polyethylene glycol (PEG) [204], polyacrylamide [205], redox-polymerized carboxymethylcellulose [206], methacrylated carboxymethylcellulose [207], poly(N-isopropylacrylamide- N,N0-dimethylacrylamide-Laponite [208], poly(acrylamide-co-acrylic acid) microhydrogels [209], poly lactide-co-glycolide [210,211], and poly glycerol monomethacrylate-poly 2-hydroxypropyl methacrylate diblock copolymer [58]. Various hybrid hydrogels have also been studied, such as PPS incorporated PEG and HA [212], a highly sulfated semi-synthetic polysaccharide combined with PEG/HA [213], poly(N-isopropylacrylamide-graft-chondroitin sulfate) hydrogels combined with or without alginate microparticles [98,214], poly D,L-lactide-co-glycolide nanoparticles carrying TGF-β3 in dextran/gelatin hydrogels [215], 1-ethyl-3(3-dimethyl aminopropyl) carbodiimide and N-hydroxysuccinimide cross-linked type II collagen/HA hydrogels [203], and nitrogen-doped plasma-polymerized ethylenes [216].

Commercial gel matrices include fiber-forming peptide gels, Hydromatrix, and Puramatrix. They were compared in terms of their potential to enable the chondrogenic differentiation of MSCs, and Hydromatrix ultimately revealed the strongest potential [217].

Growth factors, such as BMP-2, TGF-β3, GDF-5, GDF-6, and basic FGF, are combined with various materials for release [73,94,97,98,202,210,215]. Similarly, NC-CM or NP extracts with humoral factors [192,218] and chondrogenic differentiation media [219] are used in combination with various materials.

Other types of gels (non-hydrogels or undefined) that comprise natural materials—including alginate gels [56,57,220,221], poly L-lactic acid scaffolds [118], and collagen-based carriers [219,222,223]—as well as synthetic materials—including PEG diacrylate microcryogels [224], a biocompatible KLD-12 polypeptide (ACN-KLDLKLDLKLDL-CNH2)/TGF-β1 nanofiber gel [225], and layered double hydroxide nanoparticles [226]—are also all reported to be effective in the chondrogenic differentiation process of MSCs.

A comparison among the four matrices revealed that collagen, gelatin, alginate, and chitosan, in this exact order, exhibited strong potential for MSC differentiation into an NPC-like phenotype (alginate and chitosan exhibit similar potential) [227]. Another study compared alginate and chitosan hydrogels and found that alginate generated more GAGs and type II collagen [228].

Decellularized ECM—including simple decellularized NP-ECM [229,230], genipin-cross-linked decellularized NP hydrogels [231], genipin-cross-linked decellularized AF hydrogels [232], and decellularized NP and AF ECM mixtures [233]—have been demonstrated as effective scaffolds for MSCs.

The utilization of a pellet culture may allow for a similar approach. Compared to alginate beads, the pellet culture of MSCs results in higher chondrogenic differentiation [84].

A completely chimeric material, such as a silk-based scaffold, can also enhance the NP-like or AF-like differentiation of MSCs [234,235]. The majority of studies about biomaterials are observational studies confirming the MSC differentiation into NP-like/chondrogenic phenotypes by phenotypic markers or proteoglycan staining. One study utilized energy-dispersive X-ray analysis for the confirmation of proteoglycans [208]. Some studies included biomechanical analysis; however, such results are out of the scope of the present study. Briefly, biomechanical analyses include comparing the gelatin colloidal gel of various concentrations with NP tissue to find the appropriate concentration [191], a comparison of PRP and HA/PRP at different temperatures [193], a comparison of chitosan–poly(hydroxybutyrate-co-valerate) mixed with various ratios [197], and a stiffness analysis of various concentrations of agarose gel [199] or PEGs with different molecular weights [204]. None of the material that impeded the cellular viability, e.g., the high viability of MSCs seeded in chitosan hydrogel, was confirmed [196]. Table 5 summarizes the content of this section.

### 4.7. Environmental Factors

Environmental factors, including oxygen tension, osmolarity, and mechanical stress, affect MSC differentiation.

Hypoxia is a major factor determining the fate of NPCs. Since the IVD is the largest avascular tissue in vertebrates, the NP located at the center is hypoxic [236]. Generally, hypoxia confers the transcription factor hypoxia-inducible factor (HIF)-1α to function by inactivating prolyl hydroxylase and factor-inhibiting HIF, which degrades HIF-1α via proteosomal degradation [237]. The NP is a unique tissue involved in the reaction of HIF-1α partially to oxygen tension, as PHDs play a limited role in HIF-1α degradation, thereby stabilizing HIF-1α [236,238]. Nonetheless, hypoxia and HIF-1α play essential roles in the NP and in maintaining the homeostasis of tissue-controlled metabolism [239], tissue pH [240], and the NPC-like phenotype [241]. Numerous studies have reported that hypoxia promotes MSC differentiation toward an NPC-like phenotype [77,78,79,169,242,243,244]. NPC-CM treatment exerts agonistic effects on BMSC differentiation toward NPC-like cells only when cultured in hypoxia [169]. In addition, the overexpression of HIF-1α directs ADMSCs to differentiate into an NPC-like phenotype [183].

Hyperosmolarity is a characteristic feature of the NP, along with hypoxia. Negatively charged sulfated GAGs on aggrecan attract sodium ions to the NP, leading to hyperosmolarity [245]. A key molecule in this tissue, tonicity-responsive enhancer-binding protein (TonEBP/nuclear factor of activated T-cells 5 (NFAT5)), is a transcription factor recruited in a hyperosmotic environment. It suppresses the influx of sodium by regulating the intracellular levels of nonionic osmolytes such as taurine, sodium/myoinositol, and betaine, and by modulating their transporters or synthetic enzymes [245]. TonEBP also targets cyclooxygenase (COX)-2, which is well-known as an essential enzyme in prostaglandin (PG) synthesis [246]. COX-2 also plays a role in cell survival and osmoadaptation [245]. TonEBP is a positive regulator of chondroitin sulfate and aggrecan [247,248,249]. Another target of TonEBP, heat shock protein (Hsp)70, is upregulated in the NP for cell survival in harsh environments [250]. Despite the physiological hyperosmolarity of the NP, whether high osmotic pressure promotes the differentiation of MSCs into NPC-like cells remains controversial. Some studies have demonstrated positive involvement [251,252], as 400 mOsm/L pressure simulating moderate IDD exhibited an anabolic effect, and 500 mOsm/L pressure simulating healthy IVD to suppress the NP-like differentiation of ADSCs [251,253,254]. Moreover, simultaneous hypertrophy during chondrogenic differentiation of MSCs was found to depend on the type of osmolyte used [255]. However, other studies have clearly rejected any positive involvement of hyperosmolarity (pressures within the range of 400–600 mOsm/L) compared to lower osmolarity (300 mOsm/L) in NPMSCs [253] and ADMSCs [254]. Collectively, the application of hyperosmolarity to MSC differentiation toward NPC-like cells requires additional investigation.

Mechanical stresses, such as compressive strain, are loaded into IVD in daily life [7]. In a caprine organ culture model, cell viability, cell density, and gene expression were preserved with either a low dynamic compressive load (0.1–0.2 MPa, 1 Hz) or a simulated-physiological compressive load (0.1 to 0.6 MPa, a sinusoidal load with gradual change, and on/off) [7]. NPMSCs cultured in a chondrogenic differentiation medium with BMP-2 were subjected to periodic mechanical stress (0–200 kPa, 0.1 Hz), which synergistically promoted chondrogenic differentiation [70]. Multiple studies have reported similar results, including static compression [224] and cyclic and dynamic compression [256,257,258,259,260,261,262,263]. However, excessive compressive loads as high as 1.0 MPa (static) ultimately inhibit NPMSC differentiation [264]. Table 6 summarizes the content of this section.

## 5. Discussion—Highlights, Limitations, and Future Perspectives

IDD features cell loss and ECM alteration; therefore, MSC therapy is a promising strategy to regenerate cells and tissue. Numerous studies have explored novel and efficient methodologies that enable MSC differentiation in the regeneration of IVD cells.

Among the multiple types of growth factors, TGF-β superfamily members and GDF5 and 6 played the primary roles, with BMP-7 and GDF6 likely being the most effective in eliciting MSC differentiation in NPC-like cells. However, the efficacies of other endogenous and exogenous molecular factors have not been studied. Moreover, their interactions remain unknown; for example, it is not known whether these molecules synergistically enhance each other’s effects, negate them, or have completely unrelated effects. This scarcity of information is likely the limitation of their clinical use. In contrast, biomaterials and environmental factors have been extensively studied in combination with these molecules, with most studies elucidating their synergistic effects. Hence, the combination of molecules, biomaterials, and environmental factors may be helpful in identifying a novel methodology for MSC differentiation.

Numerous types of biomaterials—including natural, synthetic, and chimeric materials—have been studied; these materials simulate scaffolds of the ECM of IVD. Although synthetic and chimeric materials can be structurally sturdy, their application in clinical use is likely limited due to the likelihood of foreign body reactions [265].

Humoral factors from notochordal cells or NPCs have been studied for their role in MSC differentiation. Interestingly, the direct treatment of BMSCs with NPC exosomes was more effective than the trans-well co-culture of BMSCs with NPCs, even though the effects of the co-culture occur via exosomes. This result indicated that purified exosomes with high yield are more inductive of MSC differentiation than the generally used co-culture, suggesting that the purified exosomes are more efficient.

An important issue to address when implanting MSCs into the NP is the uniquely harsh environment of the degenerated NP. As discussed in Section 4.7 of the manuscript, the environment of the NP has low oxygen tension, acidity, relatively high osmolarity, and detrimental mechanical stress [266]. Although hypoxia improves the chondrogenic differentiation of MSCs, other factors can impede the viability of implanted MSCs. Strategies to precondition MSCs before implantation can promote cell survival. The overexpression of HIF-1α in MSCs may promote the function of monocarboxylate transporter 4 to efflux lactate from the cells or enhance the function of carbonic anhydrase 9 and 12 to recycle bicarbonate to reside in acidity [240,267]. Mechanical overload induces apoptotic cell death, which suggests that anti-apoptotic preconditioning may help MSCs to strive against such stress, including the overexpression of *BCL2* or knocking down *CASP3* [19,268].

Major risks associated with implanted cells include potential risks of tumorigenicity, immune rejection, and long-term viability of implanted cells. However, MSCs are advantageous regarding the first two concerns, as tumorigenesis has not been reported yet. Further, the privilege of IVDs to the immune system [269,270] is well-known, and no specific serological reactions were detected in a clinical trial [271], which alleviates the concern of immune rejection. MSCs reportedly can survive in porcine IVD for at least six months [32], but cellular viability thereafter is still unclear. Further study is needed to clarify this aspect, and some successive therapy may be required to maintain viable cells.

It may be inevitable for studies to compare autologous versus allogeneic MSCs. One of these two options is likely selected based on different factors, such as invasiveness, cost, and time requirements. Autologous cells are associated with fewer concerns of immunogenicity, but they are invasive to patients, especially while harvesting patient-specific cells. Moreover, they have a high cost and require a long time to increase the cell number while maintaining good clinical practice standards. In contrast, allogeneic cells can be purchased from companies in a ready-to-use form, require no invasive procedure, and have lower costs [65,272]. In addition, multiple studies have reported that no immune response occurs with allogeneic MSC implantation [65,271], which can facilitate the clinical use of allogeneic MSCs.

As a future perspective, a multifactorial perspective may be utilized to establish more advanced strategies for differentiating MSCs into NPC- or AFC-like cells. To improve the current methodologies, combining molecules, biomaterials, and environmental factors may be a promising strategy. The concentrations or molecular weights of the materials can be determined considering the importance of cellular viability and biomechanical properties. As information and discussion about the cost-effectiveness of materials are scarce, it is important to consider this when overlooking the course to clinical application. Future studies involving biomaterials should consider the cost-effectiveness of these materials. Similarly, in the stage of translational research, studies should meet the standard of good laboratory practice and undergo a thorough and general investigation to confirm the biocompatibility and safety of the materials.

Furthermore, future studies can compare multiple types of combinations to discover the best option to advance regenerative medicine for IDD. Further, a study combining these approaches should provide more advanced methodologies for IVD regeneration.

## Figures and Tables

**Table 1:** Growth factors for inducing mesenchymal stem cell differentiation into intervertebral disc cells.

Growth Factor	Effects and Examples of Usage	References
TGF-β3 (10 ng/mL [69,73,74,75]; 10 μg/mL [71])	A component of the CDM; MSC differentiation to NPC and AFC in combination with BMP-2 and IGF-1	[64,69,70,71,72,73,74,75,76]
TGF-β1 (1 ng/mL [62]; 10 ng/mL [66,78]; 20 ng/mL [77])	MSC differentiation toward the NP/chondrogenic phenotype; synergistic effect with hypoxia or GDF5; the upregulation of ERK1/2 activity; the effect is augmented with Notch 1 KD	[59,77,78,79,80,81]
BMP-2 (200 ng/mL [70]; 100 ng/mL [73]; 10 ng/mL [90])	MSC differentiation toward NPC in combination with TGF-β3, CDM, and alginate beads in a serum-free medium in combination with PRP gel	[70,73,87,90]
BMP-3 (10 ng/mL)	MSC proliferation and chondrogenic differentiation in combination with pretreatment with IL-1β	[88]
BMP-7 (100–300 ng/mL [89]; 10 ng/mL [90])	MSC differentiation toward NP-like cells via the Smad pathway; better chondrogenic differentiation potential than BMP-2	[89,90]
GDF5 (100 ng/mL [92])	MSC differentiation toward NP-like cells in combination with alginate beads	[79,91,92,93,94]
GDF6 (100 ng/mL [95,96,97,98])	MSC differentiation toward NP-like cells is better than GDF5 or TGF-β3 in combination with synthetic biomaterials	[95,96,97,98]
IGF-1 (500 ng/mL [62]; 10 ng/mL [74] 100 ng/mL [99])	MSC differentiation toward NP-like cells	[62,74,85,99]
FGF-2 (10 ng/mL [100,101])	MSC differentiation to NPC-like or chondrogenic phenotypes	[85,100,101]
PRP (platelet concentration > 1 × 10^6^/μL [82])	MSC differentiation to NPC-like or chondrogenic phenotypes; contains TGF-α and β, platelet-derived growth factors, vascular endothelial growth factor, endothelial growth factor; inferior effect compared to simple TGF-β1	[82,83,84]

AFC, annulus fibrosus cell; BMP, bone morphogenic protein; CDM, chondrogenic differentiation medium; ERK, extracellular signal-regulated kinase; FGF, fibroblast growth factor; GDF, growth differentiation factor; IGF, insulin-like growth factor; IL, interleukin; KD, knockdown; MSC, mesenchymal stem cell; NP, nucleus pulposus; NPC, nucleus pulposus cell; PRP, platelet-rich plasma; TGF, transforming growth factor.

**Table 2:** Endogenous factors for inducing mesenchymal stem cell differentiation into intervertebral disc cells.

Factors	Effects and Examples of Usage	References
Wnt3a (mouse [107]; lentiviral vector [109]; transfected L929 cells [110]; 5–40 ng/mL [108])	Controversial effects on the chondrogenic differentiation of MSCs	[105,106,107,108,109,110,111]
Wnt5a (retroviral vector [113])	Positively affected the chondrogenic differentiation of MSCs	[105,106,111,112,113]
SIRT1 (lentiviral vector)	It promotes the chondrogenic differentiation of NPMSCs by downregulating the monocyte chemoattractant protein 1 and chemokine receptor 2 axis	[117]
SOX9 (Adenoviral vector [118]; non-specified vector [122])	SOX9 transfected into BMSCs and cultivated in poly-L-lactic acid scaffolds resulted in BMSC differentiation into an NPC-like phenotype; use in combination with SIX-1 alternatively	[118,122]
Mkx (Retroviral vector)	Its overexpression resulted in MSC differentiation toward the AFC-like phenotype, possibly via the TGFβ/Smad signaling pathway	[123]
Co-Q10 (Bidepharm, 97% purification)	Hydrophobic lecithin-coated Co-Q10 protected BMSCs from oxidative stress and promoted their differentiation toward an NP-like phenotype	[124]
Link N (0.1 μg/mL or 1.0 μg/mL)	Link N alone did not induce MSC chondrogenesis in combination with CDM-induced MSC chondrogenesis	[129]
miR-15a (100 nmol/L, GenePharma)	Transfected into NPC-derived exosomes and used to treat NPMSC resulted in NPMSC chondrogenesis	[131]
miR-140-3p (Detail, NA)	Its overexpression in NPMSC facilitated cell differentiation toward the NPC-like phenotype	[133]
Serum supplementation	ADMSCs cultured without FBS have enhanced potential for chondrogenic differentiation	[135]

ADMSC, adipose-derived MSC; AFC, annulus fibrosus cell; BMSC, bone marrow-derived MSCs; CDM, chondrogenic differentiation medium; Co-Q10, Coenzyme Q10; FBS, fetal bovine serum; miR, microRNA; Mkx, Mohawk; MSCs, mesenchymal stem cells; NA, not available; NPMSC, nucleus pulposus-derived MSC; NP, nucleus pulposus; SIRT1, silent mating type information regulator 2 homolog 1; SIX-1, sine oculis homeobox homolog-1; SOX9, SRY-Box Transcription Factor 9; TGF, transforming growth factor; Wnt, Wingless signaling transduction.

**Table 3:** Exogenous factors for inducing mesenchymal stem cell differentiation into intervertebral disc cells.

Factors	Effects and Examples of Usage	References
O-vanillin (100 μM, Sigma-Aldrich, St. Louis, MO, USA)	The conditioned medium of o-vanillin-treated IVD cells induced the chondrogenic differentiation of MSCs	[138]
BSHXF (The First Hospital of Hunan University of Traditional Chinese Medicine).	ADMSCs exhibited differentiation toward an NPC-like phenotype using BSHXF-medicated serum	[140]
ASA VI (0.01–100 mg/L)	MSC differentiation into NP-like cells via regulating ERK1/2 and Smad2/3 signaling pathways	[141]
Salvianolic acid B (1–10 mg/L)	The chondrogenic differentiation of MSCs in vivo	[145]
PSO (Detail, NA)	Exerts various effects; ADMSC differentiation toward an NPC-like phenotype	[148]
Simvastatin (0.01–0.1 μM)	NPMSCs differentiate into NPC-like phenotypes following their treatments	[61]
PPS (5 μg/mL [163])	Potential to induce the chondrogenic differentiation of BMSCs	[163,164]

ADMSC, adipose-derived MSC; ASA VI, Asperosaponin VI; BMSC, bone marrow-derived MSCs; BSHXF, BuShenHuoXueFang; ERK, extracellular signal-regulated kinase; MSCs, mesenchymal stem cells; NA, not available; NPC, nucleus pulposus cell; NPMSC, nucleus pulposus-derived MSC; O-vanillin, Ortho-vanillin; PPS, pentosan polysulfate; PSO, psoralidin.

**Table 4:** Conditioned mediums, exosomes, and co-cultures for inducing mesenchymal stem cell differentiation into intervertebral disc cells.

Factors	Effects and Examples of Usage	References
CM (NCCM, 1 × 10^6^ cells/5 mL [168]; NPC-CM, not specified, P3 [169])	NCCM exhibited a stronger effect on the chondrogenic differentiation of BMSCs compared to CDM in combination with hypoxia, which is necessary for NPC-CM to induce the chondrogenic differentiation of BMSCs	[168,169]
Exosomes (1 × 10^6^ cells/5 mL [170])	NPC exosomes promote the differentiation of BMSCs into an NPC-like phenotype mediated by the Notch 1 pathway	[170,171]
Co-culture (1 × 10^6^ cells/mL, 1:1 [56]; 2 × 10^6^ cells/mL, 1:1 [57]; 1 × 10^5^ cells/0.5 cm^3^, 1:1 [75]	The co-culture of MSCs with NPCs or AFCs differentiates MSCs into NPC or AFC-like cells; the co-culture of MSCs with IVD tissue differentiates MSCs into NPC-like cells in combination with dynamic compression or bilaminar cell pellets, which have synergistic effects	[56,57,68,72,75,76,177,178,179,180,181,182,183,184,185,186,187]

AFC, annulus fibrosus cell; BMSC, bone marrow-derived MSCs; CDM, chondrogenic differentiation medium; CM, conditioned medium; IVD, intervertebral disc; MSCs, mesenchymal stem cells; NCCM, conditioned medium of notochordal cells; NPC, nucleus pulposus cell; NPC-CM, NPC-conditioned medium.

**Table 5:** Biomaterials for inducing mesenchymal stem cell differentiation into intervertebral disc cells.

Biomaterial		Differentiation	References
Hydrogel			
Natural	collagen #^1^	NP/chondrogenic	[189,190]
	gelatin #^2^		[191,192]
	HA		[193]
	alginate #^3^		[194]
	fibrin		[195]
	chitosan #^4^		[196,197,198]
	agarose		[199]
	polypeptide		[32,200]
	PRP and PRP/HA/batroxobin		[193,201]
	multiple materials combined		[202,203]
Synthetic	PEG	NP/chondrogenic	[204]
	polyacrylamide		[205]
	redox-polymerized carboxymethylcellulose		[206]
	methacrylated carboxymethylcellulose		[207]
	poly(N-isopropylacrylamide- N,N0-dimethylacrylamide-Laponite		[208]
	poly(acrylamide-co-acrylic acid) microhydrogels		[209]
	poly lactide-co-glycolide		[210,211]
	poly glycerol monomethacrylate-poly 2-hydroxypropyl methacrylate diblock copolymer		[58]
Hybrid	PPS incorporated PEG and HA	NP/chondrogenic	[212]
	a highly sulfated semi-synthetic polysaccharide combined with PEG/HA		[213]
	poly(N-isopropylacrylamide-graft-chondroitin sulfate) hydrogel combined with or without alginate microparticles		[98,214]
	poly D,L-lactide-co-glycolide nanoparticles carrying TGF-β3 in dextran/gelatin hydrogel		[215]
	1-ethyl-3(3-dimethyl aminopropyl) carbodiimide and N-hydroxysuccinimide cross-linked type II collagen/HA hydrogel		[203]
	nitrogen-doped plasma-polymerized ethylene		[216]
Commercial	Hydromatrix	NP/chondrogenic	[217]
	Puramatrix		
Other types of gels			
Natural	alginate	NP/chondrogenic	[56,57,220,221]
	poly L-lactic acid scaffolds		[118]
	collagen-based carriers		[219,222,223]
Synthetic	PEG diacrylate microcryogel		[224]
	a biocompatible KLD-12 polypeptide/TGF-β1 nanofiber gel		[225]
	layered double hydroxide nanoparticles		[226]
Other materials			
Decellularized ECM	simple decellularized NP-ECM; genipin-cross-linked decellularized NP hydrogel; genipin-cross-linked decellularized AF hydrogel; decellularized NP and AF ECM mixtures	NP/AF	[229,230,231,232,233]
Pellet culture	pellet culture of MSCs	NP/chondrogenic	[84]
Chimeric	a silk-based scaffold	NP/AF	[234,235]

AF, annulus fibrosus; HA, hyaluronic acid; NP, nucleus pulposus; PEG, polyethylene glycol; PPS, pentosan polysulfate; PRP, platelet-rich plasma; TGF, transforming growth factor; # indicates the order of potential for MSC differentiation into an NPC-like phenotype [227].

**Table 6:** Environmental factors for inducing mesenchymal stem cell differentiation into intervertebral disc cells.

Factors	Effects and Examples of Usage	References
Hypoxia	Promotes MSC differentiation toward an NPC-like phenotype; confers agonistic effects to NPC-CM treatment on BMSC differentiation toward NPC-like cells; highly relevant to HIF-1α activity	[77,78,79,169,242,243,244]
Hyperosmolarity	Whether high osmotic pressure promotes the differentiation of MSCs into NPC-like cells remains controversial	[251,252,253,254]
Mechanical stresses	A low cyclic and dynamic compressive load and a simulated-physiological compressive load promote the differentiation of MSCs into NPC-like cells	[7,70,224,256,257,258,259,260,261,262,263,264]

BMSC, bone marrow-derived MSCs; CM, conditioned medium; HIF-1α, hypoxia-inducible factor-1α; MSC, mesenchymal stem cell; NPC, nucleus pulposus cell.

## References

[1] Shapiro I.M., Vresilovic E.J., Risbud M.V. (2012). Is the spinal motion segment a diarthrodial polyaxial joint: What a nice nucleus like you doing in a joint like this?. Bone.

[2] Battié M.C., Videman T., Gibbons L.E., Fisher L.D., Manninen H., Gill K. (1995). 1995 Volvo Award in clinical sciences. Determinants of lumbar disc degeneration. A study relating lifetime exposures and magnetic resonance imaging findings in identical twins. Spine.

[3] Livshits G., Popham M., Malkin I., Sambrook P.N., Macgregor A.J., Spector T., Williams F.M.K. (2011). Lumbar disc degeneration and genetic factors are the main risk factors for low back pain in women: The UK Twin Spine Study. Ann. Rheum. Dis..

[4] Munir S., Rade M., Määttä J.H., Freidin M.B., Williams F.M.K. (2018). Intervertebral Disc Biology: Genetic Basis of Disc Degeneration. Curr. Mol. Biol. Rep..

[5] Patel A.A., Spiker W.R., Daubs M., Brodke D., Cannon-Albright L.A. (2011). Evidence for an inherited predisposition to lumbar disc disease. J. Bone Jt. Surg. Am..

[6] Williams F.M.K., Popham M., Sambrook P.N., Jones A.F., Spector T.D., MacGregor A.J. (2011). Progression of lumbar disc degeneration over a decade: A heritability study. Ann. Rheum. Dis..

[7] Paul C.P., Zuiderbaan H.A., Zandieh Doulabi B., van der Veen A.J., van de Ven P.M., Smit T.H., Helder M.N., van Royen B.J., Mullender M.G. (2012). Simulated-physiological loading conditions preserve biological and mechanical properties of caprine lumbar intervertebral discs in ex vivo culture. PLoS ONE.

[8] Paul C.P., Schoorl T., Zuiderbaan H.A., Zandieh Doulabi B., van der Veen A.J., van de Ven P.M., Smit T.H., van Royen B.J., Helder M.N., Mullender M.G. (2013). Dynamic and static overloading induce early degenerative processes in caprine lumbar intervertebral discs. PLoS ONE.

[9] Sudo H., Oda I., Abumi K., Ito M., Kotani Y., Hojo Y., Minami A. (2003). In vitro biomechanical effects of reconstruction on adjacent motion segment: Comparison of aligned/kyphotic posterolateral fusion with aligned posterior lumbar interbody fusion/posterolateral fusion. J. Neurosurg..

[10] Sudo H., Oda I., Abumi K., Ito M., Kotani Y., Minami A. (2006). Biomechanical study on the effect of five different lumbar reconstruction techniques on adjacent-level intradiscal pressure and lamina strain. J. Neurosurg. Spine.

[11] Sander A.L., Lehnert T., El Saman A., Eichler K., Marzi I., Laurer H. (2014). Outcome of traumatic intervertebral disk lesions after stabilization by internal fixator. AJR Am. J. Roentgenol..

[12] Teyssedou S., Saget M., Gayet L.E., Pries P., Breque C., Vendeuvre T. (2016). Radiologic study of disc behavior following compression fracture of the thoracolumbar hinge managed by kyphoplasty: A 52-case series. Orthop. Traumatol. Surg. Res..

[13] Toyone T., Ozawa T., Inada K., Shirahata T., Shiboi R., Watanabe A., Matsuki K., Hasue F., Fujiyoshi T., Aoki Y. (2013). Short-segment fixation without fusion for thoracolumbar burst fractures with neurological deficit can preserve thoracolumbar motion without resulting in post-traumatic disc degeneration: A 10-year follow-up study. Spine.

[14] Verlaan J.J., Dhert W.J., Oner F.C. (2013). Intervertebral disc viability after burst fractures of the thoracic and lumbar spine treated with pedicle screw fixation and direct end-plate restoration. Spine J..

[15] Wang J., Zhou Y., Zhang Z.F., Li C.Q., Zheng W.J., Liu J. (2013). Radiological study on disc degeneration of thoracolumbar burst fractures treated by percutaneous pedicle screw fixation. Eur. Spine J..

[16] Collin E.C., Carroll O., Kilcoyne M., Peroglio M., See E., Hendig D., Alini M., Grad S., Pandit A. (2017). Ageing affects chondroitin sulfates and their synthetic enzymes in the intervertebral disc. Signal Transduct. Target. Ther..

[17] Tang X., Jing L., Chen J. (2012). Changes in the molecular phenotype of nucleus pulposus cells with intervertebral disc aging. PLoS ONE.

[18] Hsieh A.H., Yoon S.T. (2010). Update on the pathophysiology of degenerative disc disease and new developments in treatment strategies. Open Access J. Sports Med..

[19] Sudo H., Minami A. (2011). Caspase 3 as a therapeutic target for regulation of intervertebral disc degeneration in rabbits. Arthritis Rheum..

[20] Ohnishi T., Yamada K., Iwasaki K., Tsujimoto T., Higashi H., Kimura T., Iwasaki N., Sudo H. (2019). Caspase-3 knockout inhibits intervertebral disc degeneration related to injury but accelerates degeneration related to aging. Sci. Rep..

[21] Shin E.H., Cho K.J., Kim Y.T., Park M.H. (2018). Risk factors for recurrent lumbar disc herniation after discectomy. Int. Orthop..

[22] Hilibrand A.S., Carlson G.D., Palumbo M.A., Jones P.K., Bohlman H.H. (1999). Radiculopathy and myelopathy at segments adjacent to the site of a previous anterior cervical arthrodesis. J. Bone Jt. Surg. Am..

[23] Melrose J. (2016). Strategies in regenerative medicine for intervertebral disc repair using mesenchymal stem cells and bioscaffolds. Regen. Med..

[24] Bertolo A., Thiede T., Aebli N., Baur M., Ferguson S.J., Stoyanov J.V. (2011). Human mesenchymal stem cell co-culture modulates the immunological properties of human intervertebral disc tissue fragments in vitro. Eur. Spine J..

[25] Arkesteijn I.T., Smolders L.A., Spillekom S., Riemers F.M., Potier E., Meij B.P., Ito K., Tryfonidou M.A. (2015). Effect of coculturing canine notochordal, nucleus pulposus and mesenchymal stromal cells for intervertebral disc regeneration. Arthritis Res. Ther..

[26] Neidlinger-Wilke C., Ekkerlein A., Goncalves R.M., Ferreira J.R., Ignatius A., Wilke H.J., Teixeira G.Q. (2021). Mesenchymal stem cell secretome decreases the inflammatory response in annulus fibrosus organ cultures. Eur. Cell Mater..

[27] Shim E.K., Lee J.S., Kim D.E., Kim S.K., Jung B.J., Choi E.Y., Kim C.S. (2016). Autogenous Mesenchymal Stem Cells from the Vertebral Body Enhance Intervertebral Disc Regeneration via Paracrine Interaction: An in Vitro Pilot Study. Cell Transplant..

[28] Svanvik T., Henriksson H.B., Karlsson C., Hagman M., Lindahl A., Brisby H. (2010). Human disk cells from degenerated disks and mesenchymal stem cells in co-culture result in increased matrix production. Cells Tissues Organs.

[29] Yang S.H., Wu C.C., Shih T.T., Sun Y.H., Lin F.H. (2008). In vitro study on interaction between human nucleus pulposus cells and mesenchymal stem cells through paracrine stimulation. Spine.

[30] Yang F., Leung V.Y., Luk K.D., Chan D., Cheung K.M. (2009). Mesenchymal stem cells arrest intervertebral disc degeneration through chondrocytic differentiation and stimulation of endogenous cells. Mol. Ther..

[31] Liang C., Li H., Tao Y., Zhou X., Li F., Chen G., Chen Q. (2012). Responses of human adipose-derived mesenchymal stem cells to chemical microenvironment of the intervertebral disc. J. Transl. Med..

[32] Henriksson H.B., Svanvik T., Jonsson M., Hagman M., Horn M., Lindahl A., Brisby H. (2009). Transplantation of human mesenchymal stems cells into intervertebral discs in a xenogeneic porcine model. Spine.

[33] Mochida J., Sakai D., Nakamura Y., Watanabe T., Yamamoto Y., Kato S. (2015). Intervertebral disc repair with activated nucleus pulposus cell transplantation: A three-year, prospective clinical study of its safety. Eur. Cell Mater..

[34] Meisel H.J., Ganey T., Hutton W.C., Libera J., Minkus Y., Alasevic O. (2006). Clinical experience in cell-based therapeutics: Intervention and outcome. Eur. Spine J..

[35] Meisel H.J., Siodla V., Ganey T., Minkus Y., Hutton W.C., Alasevic O.J. (2007). Clinical experience in cell-based therapeutics: Disc chondrocyte transplantation A treatment for degenerated or damaged intervertebral disc. Biomol. Eng..

[36] Yang W., Yu X.H., Wang C., He W.S., Zhang S.J., Yan Y.G., Zhang J., Xiang Y.X., Wang W.J. (2015). Interleukin-1β in intervertebral disk degeneration. Clin. Chim. Acta.

[37] Suzuki S., Fujita N., Fujii T., Watanabe K., Yagi M., Tsuji T., Ishii K., Miyamoto T., Horiuchi K., Nakamura M. (2017). Potential Involvement of the IL-6/JAK/STAT3 Pathway in the Pathogenesis of Intervertebral Disc Degeneration. Spine.

[38] Wang C., Yu X., Yan Y., Yang W., Zhang S., Xiang Y., Zhang J., Wang W. (2017). Tumor necrosis factor-α: A key contributor to intervertebral disc degeneration. Acta Biochim. Biophys. Sin..

[39] Zhao C.Q., Liu D., Li H., Jiang L.S., Dai L.Y. (2007). Interleukin-1beta enhances the effect of serum deprivation on rat annular cell apoptosis. Apoptosis.

[40] Yamada K., Sudo H., Iwasaki K., Sasaki N., Higashi H., Kameda Y., Ito M., Takahata M., Abumi K., Minami A. (2014). Caspase 3 silencing inhibits biomechanical overload-induced intervertebral disk degeneration. Am. J. Pathol..

[41] Mohanty S., Pinelli R., Pricop P., Albert T.J., Dahia C.L. (2019). Chondrocyte-like nested cells in the aged intervertebral disc are late-stage nucleus pulposus cells. Aging cell.

[42] Choi H., Tessier S., Silagi E.S., Kyada R., Yousefi F., Pleshko N., Shapiro I.M., Risbud M.V. (2018). A novel mouse model of intervertebral disc degeneration shows altered cell fate and matrix homeostasis. Matrix Biol..

[43] Ohnishi T., Iwasaki N., Sudo H. (2022). Causes of and Molecular Targets for the Treatment of Intervertebral Disc Degeneration: A Review. Cells.

[44] Ohnishi T., Novais E.J., Risbud M.V. (2020). Alterations in ECM signature underscore multiple sub-phenotypes of intervertebral disc degeneration. Matrix Biol. Plus.

[45] Novais E.J., Diekman B.O., Shapiro I.M., Risbud M.V. (2019). p16(Ink4a) deletion in cells of the intervertebral disc affects their matrix homeostasis and senescence associated secretory phenotype without altering onset of senescence. Matrix Biol..

[46] Cherif H., Bisson D.G., Mannarino M., Rabau O., Ouellet J.A., Haglund L. (2020). Senotherapeutic drugs for human intervertebral disc degeneration and low back pain. Elife.

[47] Shi P.Z., Wang J.W., Wang P.C., Han B., Lu X.H., Ren Y.X., Feng X.M., Cheng X.F., Zhang L. (2021). Urolithin a alleviates oxidative stress-induced senescence in nucleus pulposus-derived mesenchymal stem cells through SIRT1/PGC-1α pathway. World J. Stem Cells.

[48] Xu X., Wang D., Zheng C., Gao B., Fan J., Cheng P., Liu B., Yang L., Luo Z. (2019). Progerin accumulation in nucleus pulposus cells impairs mitochondrial function and induces intervertebral disc degeneration and therapeutic effects of sulforaphane. Theranostics.

[49] Wang F., Cai F., Shi R., Wang X.H., Wu X.T. (2016). Aging and age related stresses: A senescence mechanism of intervertebral disc degeneration. Osteoarthr. Cartil..

[50] Krock E., Rosenzweig D.H., Chabot-Dore A.J., Jarzem P., Weber M.H., Ouellet J.A., Stone L.S., Haglund L. (2014). Painful, degenerating intervertebral discs up-regulate neurite sprouting and CGRP through nociceptive factors. J. Cell Mol. Med..

[51] Esquijarosa Hechavarria M., Richard S.A. (2022). Edifying the Focal Factors Influencing Mesenchymal Stem Cells by the Microenvironment of Intervertebral Disc Degeneration in Low Back Pain. Pain. Res. Manag..

[52] Jackson W.M., Nesti L.J., Tuan R.S. (2012). Concise review: Clinical translation of wound healing therapies based on mesenchymal stem cells. Stem Cells Transl. Med..

[53] Zeng X., Lin J., Wu H., Yu J., Tu M., Cheang L.H., Zhang J. (2020). Effect of Conditioned Medium from Human Umbilical Cord-Derived Mesenchymal Stromal Cells on Rejuvenation of Nucleus Pulposus Derived Stem/Progenitor Cells from Degenerated Intervertebral Disc. Int. J. Stem Cells.

[54] Lehmann T.P., Filipiak K., Juzwa W., Sujka-Kordowska P., Jagodziński P.P., Zabel M., Głowacki J., Misterska E., Walczak M., Głowacki M. (2014). Co-culture of human nucleus pulposus cells with multipotent mesenchymal stromal cells from human bone marrow reveals formation of tunnelling nanotubes. Mol. Med. Rep..

[55] Sun Z., Mi C. (2023). On the identification of the ultra-structural organization of elastic fibers and their effects on the integrity of annulus fibrosus. J. Biomech..

[56] Ukeba D., Sudo H., Tsujimoto T., Ura K., Yamada K., Iwasaki N. (2020). Bone marrow mesenchymal stem cells combined with ultra-purified alginate gel as a regenerative therapeutic strategy after discectomy for degenerated intervertebral discs. EBioMedicine.

[57] Ukeba D., Yamada K., Suyama T., Lebl D.R., Tsujimoto T., Nonoyama T., Sugino H., Iwasaki N., Watanabe M., Matsuzaki Y. (2022). Combination of ultra-purified stem cells with an in situ-forming bioresorbable gel enhances intervertebral disc regeneration. EBioMedicine.

[58] Binch A.L.A., Ratcliffe L.P.D., Milani A.H., Saunders B.R., Armes S.P., Hoyland J.A. (2021). Site-Directed Differentiation of Human Adipose-Derived Mesenchymal Stem Cells to Nucleus Pulposus Cells Using an Injectable Hydroxyl-Functional Diblock Copolymer Worm Gel. Biomacromolecules.

[59] Colombier P., Clouet J., Boyer C., Ruel M., Bonin G., Lesoeur J., Moreau A., Fellah B.H., Weiss P., Lescaudron L. (2016). TGF-β1 and GDF5 Act Synergistically to Drive the Differentiation of Human Adipose Stromal Cells toward Nucleus Pulposus-like Cells. Stem Cells.

[60] Zhang H., Ma X., Zhang L., Guan X., Bai T., Xue C. (2015). The ability to form cartilage of NPMSC and BMSC in SD rats. Int. J. Clin. Exp. Med..

[61] Huang Z., Cheng X., Zhao J., Liu Z., Wang J., Feng X., Zhang L. (2019). Influence of simvastatin on the biological behavior of nucleus pulposus-derived mesenchymal stem cells. Iran. J. Basic. Med. Sci..

[62] Chon B.H., Lee E.J., Jing L., Setton L.A., Chen J. (2013). Human umbilical cord mesenchymal stromal cells exhibit immature nucleus pulposus cell phenotype in a laminin-rich pseudo-three-dimensional culture system. Stem Cell Res. Ther..

[63] Gou S., Oxentenko S.C., Eldrige J.S., Xiao L., Pingree M.J., Wang Z., Perez-Terzic C., Qu W. (2014). Stem cell therapy for intervertebral disk regeneration. Am. J. Phys. Med. Rehabil..

[64] Ekram S., Khalid S., Bashir I., Salim A., Khan I. (2021). Human umbilical cord-derived mesenchymal stem cells and their chondroprogenitor derivatives reduced pain and inflammation signaling and promote regeneration in a rat intervertebral disc degeneration model. Mol. Cell Biochem..

[65] Lewandrowski K.U., Dowling A., Vera J.C., Leon J.F.R., Telfeian A.E., Lorio M.P. (2023). Pain Relief After Allogenic Stem Cell Disc Therapy. Pain. Physician.

[66] Dai X., Guan Y., Zhang Z., Xiong Y., Liu C., Li H., Liu B. (2021). Comparison of the differentiation abilities of bone marrow-derived mesenchymal stem cells and adipose-derived mesenchymal stem cells toward nucleus pulposus-like cells in three-dimensional culture. Exp. Ther. Med..

[67] Vadalà G., Sowa G., Hubert M., Gilbertson L.G., Denaro V., Kang J.D. (2012). Mesenchymal stem cells injection in degenerated intervertebral disc: Cell leakage may induce osteophyte formation. J. Tissue Eng. Regen. Med..

[68] Zhou Y., Hu X., Zheng X., Wu Y., Tian N., Xu H., Zhang X. (2017). Differentiation Potential of Mesenchymal Stem Cells Derived from Adipose Tissue vs. Bone Marrow Toward Annulus Fibrosus Cells In vitro. Curr. Stem Cell Res. Ther..

[69] Okita N., Honda Y., Kishimoto N., Liao W., Azumi E., Hashimoto Y., Matsumoto N. (2015). Supplementation of strontium to a chondrogenic medium promotes chondrogenic differentiation of human dedifferentiated fat cells. Tissue Eng. Part A.

[70] Liu Y., Gao G.M., Yang K.Y., Nong L.M. (2022). Construction of tissue-engineered nucleus pulposus by stimulation with periodic mechanical stress and BMP-2. iScience.

[71] Steck E., Bertram H., Abel R., Chen B., Winter A., Richter W. (2005). Induction of intervertebral disc-like cells from adult mesenchymal stem cells. Stem Cells.

[72] Jin E.S., Min J., Jeon S.R., Choi K.H., Jeong J.H. (2013). Analysis of molecular expression in adipose tissue-derived mesenchymal stem cells: Prospects for use in the treatment of intervertebral disc degeneration. J. Korean Neurosurg. Soc..

[73] Shen B., Wei A., Tao H., Diwan A.D., Ma D.D. (2009). BMP-2 enhances TGF-beta3-mediated chondrogenic differentiation of human bone marrow multipotent mesenchymal stromal cells in alginate bead culture. Tissue Eng. Part A.

[74] Tao Y., Zhou X., Liang C., Li H., Han B., Li F., Chen Q. (2015). TGF-β3 and IGF-1 synergy ameliorates nucleus pulposus mesenchymal stem cell differentiation towards the nucleus pulposus cell type through MAPK/ERK signaling. Growth Factors.

[75] Gruber H.E., Deepe R., Hoelscher G.L., Ingram J.A., Norton H.J., Scannell B., Loeffler B.J., Zinchenko N., Hanley E.N., Tapp H. (2010). Human adipose-derived mesenchymal stem cells: Direction to a phenotype sharing similarities with the disc, gene expression profiling, and coculture with human annulus cells. Tissue Eng. Part A.

[76] Tapp H., Deepe R., Ingram J.A., Kuremsky M., Hanley E.N., Gruber H.E. (2008). Adipose-derived mesenchymal stem cells from the sand rat: Transforming growth factor beta and 3D co-culture with human disc cells stimulate proteoglycan and collagen type I rich extracellular matrix. Arthritis Res. Ther..

[77] Feng G., Jin X., Hu J., Ma H., Gupte M.J., Liu H., Ma P.X. (2011). Effects of hypoxias and scaffold architecture on rabbit mesenchymal stem cell differentiation towards a nucleus pulposus-like phenotype. Biomaterials.

[78] Risbud M.V., Albert T.J., Guttapalli A., Vresilovic E.J., Hillibrand A.S., Vaccaro A.R., Shapiro I.M. (2004). Differentiation of mesenchymal stem cells towards a nucleus pulposus-like phenotype in vitro: Implications for cell-based transplantation therapy. Spine.

[79] Stoyanov J.V., Gantenbein-Ritter B., Bertolo A., Aebli N., Baur M., Alini M., Grad S. (2011). Role of hypoxia and growth and differentiation factor-5 on differentiation of human mesenchymal stem cells towards intervertebral nucleus pulposus-like cells. Eur. Cell Mater..

[80] Morigele M., Shao Z., Zhang Z., Kaige M., Zhang Y., Qiang W., Yang S. (2013). TGF-β1 induces a nucleus pulposus-like phenotype in Notch 1 knockdown rabbit bone marrow mesenchymal stem cells. Cell Biol. Int..

[81] Han C., Jiang C., Yu C., Shen H. (2015). Differentiation of transforming growth factor β1-induced mesenchymal stem cells into nucleus pulposus-like cells under simulated microgravity conditions. Cell Mol. Biol..

[82] Everts P., Onishi K., Jayaram P., Lana J.F., Mautner K. (2020). Platelet-Rich Plasma: New Performance Understandings and Therapeutic Considerations in 2020. Int. J. Mol. Sci..

[83] Jia J., Wang S.Z., Ma L.Y., Yu J.B., Guo Y.D., Wang C. (2018). The Differential Effects of Leukocyte-Containing and Pure Platelet-Rich Plasma on Nucleus Pulposus-Derived Mesenchymal Stem Cells: Implications for the Clinical Treatment of Intervertebral Disc Degeneration. Stem Cells Int..

[84] Mietsch A., Neidlinger-Wilke C., Schrezenmeier H., Mauer U.M., Friemert B., Wilke H.J., Ignatius A. (2013). Evaluation of platelet-rich plasma and hydrostatic pressure regarding cell differentiation in nucleus pulposus tissue engineering. J. Tissue Eng. Regen. Med..

[85] Ehlicke F., Freimark D., Heil B., Dorresteijn A., Czermak P. (2010). Intervertebral disc regeneration: Influence of growth factors on differentiation of human mesenchymal stem cells (hMSC). Int. J. Artif. Organs.

[86] Chen G., Deng C., Li Y.P. (2012). TGF-β and BMP signaling in osteoblast differentiation and bone formation. Int. J. Biol. Sci..

[87] Hou Y., Shi G., Shi J., Xu G., Guo Y., Xu P. (2016). Study design: In vitro and in vivo assessment of bone morphogenic protein 2 combined with platelet-rich plasma on treatment of disc degeneration. Int. Orthop..

[88] Hingert D., Barreto Henriksson H., Brisby H. (2018). Human Mesenchymal Stem Cells Pretreated with Interleukin-1β and Stimulated with Bone Morphogenetic Growth Factor-3 Enhance Chondrogenesis. Tissue Eng. Part A.

[89] Xu J., E X.Q., Wang N.X., Wang M.N., Xie H.X., Cao Y.H., Sun L.H., Tian J., Chen H.J., Yan J.L. (2016). BMP7 enhances the effect of BMSCs on extracellular matrix remodeling in a rabbit model of intervertebral disc degeneration. FEBS J..

[90] Knippenberg M., Helder M.N., Zandieh Doulabi B., Wuisman P.I., Klein-Nulend J. (2006). Osteogenesis versus chondrogenesis by BMP-2 and BMP-7 in adipose stem cells. Biochem. Biophys. Res. Commun..

[91] Zhu K., Zhao R., Ye Y., Xu G., Zhang C. (2022). Effect of lentivirus-mediated growth and differentiation factor-5 transfection on differentiation of rabbit nucleus pulposus mesenchymal stem cells. Eur. J. Med. Res..

[92] Gantenbein-Ritter B., Benneker L.M., Alini M., Grad S. (2011). Differential response of human bone marrow stromal cells to either TGF-β(1) or rhGDF-5. Eur. Spine J..

[93] Feng C., Liu H., Yang Y., Huang B., Zhou Y. (2015). Growth and differentiation factor-5 contributes to the structural and functional maintenance of the intervertebral disc. Cell Physiol. Biochem..

[94] Bucher C., Gazdhar A., Benneker L.M., Geiser T., Gantenbein-Ritter B. (2013). Nonviral Gene Delivery of Growth and Differentiation Factor 5 to Human Mesenchymal Stem Cells Injected into a 3D Bovine Intervertebral Disc Organ Culture System. Stem Cells Int..

[95] Hodgkinson T., Wignall F., Hoyland J.A., Richardson S.M. (2020). High BMPR2 expression leads to enhanced SMAD1/5/8 signalling and GDF6 responsiveness in human adipose-derived stem cells: Implications for stem cell therapies for intervertebral disc degeneration. J. Tissue Eng..

[96] Clarke L.E., McConnell J.C., Sherratt M.J., Derby B., Richardson S.M., Hoyland J.A. (2014). Growth differentiation factor 6 and transforming growth factor-beta differentially mediate mesenchymal stem cell differentiation, composition, and micromechanical properties of nucleus pulposus constructs. Arthritis Res. Ther..

[97] Hodgkinson T., Stening J.Z., White L.J., Shakesheff K.M., Hoyland J.A., Richardson S.M. (2019). Microparticles for controlled growth differentiation factor 6 delivery to direct adipose stem cell-based nucleus pulposus regeneration. J. Tissue Eng. Regen. Med..

[98] Christiani T., Mys K., Dyer K., Kadlowec J., Iftode C., Vernengo A.J. (2021). Using embedded alginate microparticles to tune the properties of in situ forming poly(N-isopropylacrylamide)-graft-chondroitin sulfate bioadhesive hydrogels for replacement and repair of the nucleus pulposus of the intervertebral disc. JOR Spine.

[99] Longobardi L., O’Rear L., Aakula S., Johnstone B., Shimer K., Chytil A., Horton W.A., Moses H.L., Spagnoli A. (2006). Effect of IGF-I in the chondrogenesis of bone marrow mesenchymal stem cells in the presence or absence of TGF-beta signaling. J. Bone Miner. Res..

[100] Tsai T.T., Guttapalli A., Oguz E., Chen L.H., Vaccaro A.R., Albert T.J., Shapiro I.M., Risbud M.V. (2007). Fibroblast growth factor-2 maintains the differentiation potential of nucleus pulposus cells in vitro: Implications for cell-based transplantation therapy. Spine.

[101] Chiou M., Xu Y., Longaker M.T. (2006). Mitogenic and chondrogenic effects of fibroblast growth factor-2 in adipose-derived mesenchymal cells. Biochem. Biophys. Res. Commun..

[102] Zhou X., Tao Y., Wang J., Liang C., Wang J., Li H., Chen Q. (2015). Roles of FGF-2 and TGF-beta/FGF-2 on differentiation of human mesenchymal stem cells towards nucleus pulposus-like phenotype. Growth Factors.

[103] Chen Y., Alman B.A. (2009). Wnt pathway, an essential role in bone regeneration. J. Cell Biochem..

[104] Akiyama T. (2000). Wnt/beta-catenin signaling. Cytokine Growth Factor. Rev..

[105] Volleman T.N.E., Schol J., Morita K., Sakai D., Watanabe M. (2020). Wnt3a and wnt5a as Potential Chondrogenic Stimulators for Nucleus Pulposus Cell Induction: A Comprehensive Review. Neurospine.

[106] Pei M., Li J., Zhang Y., Liu G., Wei L., Zhang Y. (2014). Expansion on a matrix deposited by nonchondrogenic urine stem cells strengthens the chondrogenic capacity of repeated-passage bone marrow stromal cells. Cell Tissue Res..

[107] Fischer L., Boland G., Tuan R.S. (2002). Wnt-3A enhances bone morphogenetic protein-2-mediated chondrogenesis of murine C3H10T1/2 mesenchymal cells. J. Biol. Chem..

[108] Centola M., Tonnarelli B., Schären S., Glaser N., Barbero A., Martin I. (2013). Priming 3D cultures of human mesenchymal stromal cells toward cartilage formation via developmental pathways. Stem Cells Dev..

[109] Qu F., Wang J., Xu N., Liu C., Li S., Wang N., Qi W., Li H., Li C., Geng Z. (2013). WNT3A modulates chondrogenesis via canonical and non-canonical Wnt pathways in MSCs. Front. Biosci..

[110] Hwang S.G., Yu S.S., Lee S.W., Chun J.S. (2005). Wnt-3a regulates chondrocyte differentiation via c-Jun/AP-1 pathway. FEBS Lett..

[111] Hsu S.H., Huang G.S. (2013). Substrate-dependent Wnt signaling in MSC differentiation within biomaterial-derived 3D spheroids. Biomaterials.

[112] Dickinson S.C., Sutton C.A., Brady K., Salerno A., Katopodi T., Williams R.L., West C.C., Evseenko D., Wu L., Pang S. (2017). The Wnt5a Receptor, Receptor Tyrosine Kinase-Like Orphan Receptor 2, Is a Predictive Cell Surface Marker of Human Mesenchymal Stem Cells with an Enhanced Capacity for Chondrogenic Differentiation. Stem Cells.

[113] Church V., Nohno T., Linker C., Marcelle C., Francis-West P. (2002). Wnt regulation of chondrocyte differentiation. J. Cell Sci..

[114] Zhu Z., Xing H., Tang R., Qian S., He S., Hu Q., Zhang N. (2021). The preconditioning of lithium promotes mesenchymal stem cell-based therapy for the degenerated intervertebral disc via upregulating cellular ROS. Stem Cell Res. Ther..

[115] Wang X., Li H., Xu K., Zhu H., Peng Y., Liang A., Li C., Huang D., Ye W. (2016). SIRT1 expression is refractory to hypoxia and inflammatory cytokines in nucleus pulposus cells: Novel regulation by HIF-1α and NF-κB signaling. Cell Biol. Int..

[116] Zhang Z., Lin J., Nisar M., Chen T., Xu T., Zheng G., Wang C., Jin H., Chen J., Gao W. (2019). The Sirt1/P53 Axis in Diabetic Intervertebral Disc Degeneration Pathogenesis and Therapeutics. Oxid. Med. Cell Longev..

[117] Ou X., Ying J., Bai X., Wang C., Ruan D. (2020). Activation of SIRT1 promotes cartilage differentiation and reduces apoptosis of nucleus pulposus mesenchymal stem cells via the MCP1/CCR2 axis in subjects with intervertebral disc degeneration. Int. J. Mol. Med..

[118] Richardson S.M., Curran J.M., Chen R., Vaughan-Thomas A., Hunt J.A., Freemont A.J., Hoyland J.A. (2006). The differentiation of bone marrow mesenchymal stem cells into chondrocyte-like cells on poly-L-lactic acid (PLLA) scaffolds. Biomaterials.

[119] Tsingas M., Ottone O.K., Haseeb A., Barve R.A., Shapiro I.M., Lefebvre V., Risbud M.V. (2020). Sox9 deletion causes severe intervertebral disc degeneration characterized by apoptosis, matrix remodeling, and compartment-specific transcriptomic changes. Matrix Biol..

[120] Oliver G., Wehr R., Jenkins N.A., Copeland N.G., Cheyette B.N., Hartenstein V., Zipursky S.L., Gruss P. (1995). Homeobox genes and connective tissue patterning. Development.

[121] Zhu L., Jiang S., Yu S., Liu X., Pu S., Xie P., Chen H., Liao X., Wang K., Wang B. (2020). Increased SIX-1 expression promotes breast cancer metastasis by regulating lncATB-miR-200s-ZEB1 axis. J. Cell Mol. Med..

[122] Khalid S., Ekram S., Salim A., Chaudhry G.R., Khan I. (2022). Transcription regulators differentiate mesenchymal stem cells into chondroprogenitors, and their in vivo implantation regenerated the intervertebral disc degeneration. World J. Stem Cells.

[123] Nakamichi R., Ito Y., Inui M., Onizuka N., Kayama T., Kataoka K., Suzuki H., Mori M., Inagawa M., Ichinose S. (2016). Mohawk promotes the maintenance and regeneration of the outer annulus fibrosus of intervertebral discs. Nature Commun..

[124] Sun J., Yang F., Wang L., Yu H., Yang Z., Wei J., Vasilev K., Zhang X., Liu X., Zhao Y. (2023). Delivery of coenzyme Q10 loaded micelle targets mitochondrial ROS and enhances efficiency of mesenchymal stem cell therapy in intervertebral disc degeneration. Bioact. Mater..

[125] Wang Y., Hekimi S. (2016). Understanding Ubiquinone. Trends Cell Biol..

[126] Gutierrez-Mariscal F.M., Arenas-de Larriva A.P., Limia-Perez L., Romero-Cabrera J.L., Yubero-Serrano E.M., López-Miranda J. (2020). Coenzyme Q(10) Supplementation for the Reduction of Oxidative Stress: Clinical Implications in the Treatment of Chronic Diseases. Int. J. Mol. Sci..

[127] Petit A., Yao G., Rowas S.A., Gawri R., Epure L., Antoniou J., Mwale F. (2011). Effect of synthetic link N peptide on the expression of type I and type II collagens in human intervertebral disc cells. Tissue Eng. Part A.

[128] Mwale F., Demers C.N., Petit A., Roughley P., Poole A.R., Steffen T., Aebi M., Antoniou J. (2003). A synthetic peptide of link protein stimulates the biosynthesis of collagens II, IX and proteoglycan by cells of the intervertebral disc. J. Cell Biochem..

[129] Antoniou J., Wang H.T., Alaseem A.M., Haglund L., Roughley P.J., Mwale F. (2012). The effect of Link N on differentiation of human bone marrow-derived mesenchymal stem cells. Arthritis Res. Ther..

[130] Cai P., Yang T., Jiang X., Zheng M., Xu G., Xia J. (2017). Role of miR-15a in intervertebral disc degeneration through targeting MAP3K9. Biomed. Pharmacother..

[131] Zhang Q., Shen Y., Zhao S., Jiang Y., Zhou D., Zhang Y. (2021). Exosomes miR-15a promotes nucleus pulposus-mesenchymal stem cells chondrogenic differentiation by targeting MMP-3. Cell Signal.

[132] Zhang Q., Weng Y., Jiang Y., Zhao S., Zhou D., Xu N. (2018). Overexpression of miR-140-5p inhibits lipopolysaccharide-induced human intervertebral disc inflammation and degeneration by downregulating toll-like receptor 4. Oncol. Rep..

[133] Wang Z., Zhang S., Zhao Y., Qu Z., Zhuang X., Song Q., Leng J., Liu Y. (2021). MicroRNA-140-3p alleviates intervertebral disc degeneration via KLF5/N-cadherin/MDM2/Slug axis. RNA Biol..

[134] Brunner D., Frank J., Appl H., Schöffl H., Pfaller W., Gstraunthaler G. (2010). Serum-free cell culture: The serum-free media interactive online database. Altex.

[135] Wan Safwani W.K., Wong C.W., Yong K.W., Choi J.R., Mat Adenan N.A., Omar S.Z., Wan Abas W.A., Pingguan-Murphy B. (2016). The effects of hypoxia and serum-free conditions on the stemness properties of human adipose-derived stem cells. Cytotechnology.

[136] Nasr S., Varshosaz J., Hajhashemi V. (2020). Ortho-vanillin nanoparticle-doped glucan microspheres exacerbate the anti-arthritic effects of methotrexate in adjuvant-induced arthritis in rats. Pharmacol. Rep..

[137] Cherif H., Bisson D.G., Jarzem P., Weber M., Ouellet J.A., Haglund L. (2019). Curcumin and o-Vanillin Exhibit Evidence of Senolytic Activity in Human IVD Cells In Vitro. J. Clin. Med..

[138] Li L., Sheng K., Mannarino M., Jarzem P., Cherif H., Haglund L. (2022). o-Vanillin Modulates Cell Phenotype and Extracellular Vesicles of Human Mesenchymal Stem Cells and Intervertebral Disc Cells. Cells.

[139] Yang S., Li L., Zhu L., Zhang C., Li Z., Guo Y., Nie Y., Luo Z. (2019). Bu-Shen-Huo-Xue-Fang modulates nucleus pulposus cell proliferation and extracellular matrix remodeling in intervertebral disk degeneration through miR-483 regulation of Wnt pathway. J. Cell Biochem..

[140] Duan J., Li Z., Liu E., Long H., Chen L., Yang S. (2023). BSHXF-medicated serum combined with ADSCs regulates the TGF-β1/Smad pathway to repair oxidatively damaged NPCs and its component analysis. J. Ethnopharmacol..

[141] Niu Y.T., Xie L., Deng R.R., Zhang X.Y. (2021). In the presence of TGF-β1, Asperosaponin VI promotes human mesenchymal stem cell differentiation into nucleus pulposus like- cells. BMC Complement. Med. Ther..

[142] Niu Y., Li Y., Huang H., Kong X., Zhang R., Liu L., Sun Y., Wang T., Mei Q. (2011). Asperosaponin VI, a saponin component from Dipsacus asper wall, induces osteoblast differentiation through bone morphogenetic protein-2/p38 and extracellular signal-regulated kinase 1/2 pathway. Phytother. Res..

[143] Ho J.H., Hong C.Y. (2011). Salvianolic acids: Small compounds with multiple mechanisms for cardiovascular protection. J. Biomed. Sci..

[144] Ren J., Fu L., Nile S.H., Zhang J., Kai G. (2019). Salvia miltiorrhiza in Treating Cardiovascular Diseases: A Review on Its Pharmacological and Clinical Applications. Front. Pharmacol..

[145] Yan H.S., Hang C., Chen S.W., Wang K.K., Bo P. (2020). Salvianolic acid B combined with mesenchymal stem cells contributes to nucleus pulposus regeneration. Connect. Tissue Res..

[146] Sharifi-Rad J., Kamiloglu S., Yeskaliyeva B., Beyatli A., Alfred M.A., Salehi B., Calina D., Docea A.O., Imran M., Anil Kumar N.V. (2020). Pharmacological Activities of Psoralidin: A Comprehensive Review of the Molecular Mechanisms of Action. Front. Pharmacol..

[147] Zhang R., Shi W., Li L., Huang X., Xu D., Wu L. (2019). Biological activity and health promoting effects of psoralidin. Pharmazie.

[148] Li S., Liu X., Nie Y., Yang L., Zhang C., Guo Y., Yang S., Li Z. (2023). Psoralidin Induced Differentiation from Adipose-derived Stem Cells to Nucleus Pulposus-like Cells by TGF-β/Smad Signaling. Curr. Mol. Med..

[149] Tu J., Li W., Zhang Y., Wu X., Song Y., Kang L., Liu W., Wang K., Li S., Hua W. (2017). Simvastatin Inhibits IL-1β-Induced Apoptosis and Extracellular Matrix Degradation by Suppressing the NF-kB and MAPK Pathways in Nucleus Pulposus Cells. Inflammation.

[150] Zhang H., Lin C.Y. (2008). Simvastatin stimulates chondrogenic phenotype of intervertebral disc cells partially through BMP-2 pathway. Spine.

[151] Niu J., Ding G., Zhang L. (2015). Effects of simvastatin on the osteogenic differentiation and immunomodulation of bone marrow mesenchymal stem cells. Mol. Med. Rep..

[152] Bing W., Pang X., Qu Q., Bai X., Yang W., Bi Y., Bi X. (2016). Simvastatin improves the homing of BMSCs via the PI3K/AKT/miR-9 pathway. J. Cell Mol. Med..

[153] Smith M.M., Melrose J. (2023). Pentosan Polysulfate Affords Pleotropic Protection to Multiple Cells and Tissues. Pharmaceuticals.

[154] Teichman J.M. (2002). The role of pentosan polysulfate in treatment approaches for interstitial cystitis. Rev. Urol..

[155] Klegeris A., Singh E.A., McGeer P.L. (2002). Effects of C-reactive protein and pentosan polysulphate on human complement activation. Immunology.

[156] Orme C.E., Harris R.C. (1997). A comparison of the lipolytic and anticoagulative properties of heparin and pentosan polysulphate in the thoroughbred horse. Acta Physiol. Scand..

[157] Vinazzer H. (1991). Effect of pentosan polysulfate on fibrinolysis: Basic tests and clinical application. Semin. Thromb. Hemost..

[158] Tardy-Poncet B., Tardy B., Grelac F., Reynaud J., Mismetti P., Bertrand J.C., Guyotat D. (1994). Pentosan polysulfate-induced thrombocytopenia and thrombosis. Am. J. Hematol..

[159] Francis D.J., Hutadilok N., Kongtawelert P., Ghosh P. (1993). Pentosan polysulphate and glycosaminoglycan polysulphate stimulate the synthesis of hyaluronan in vivo. Rheumatol. Int..

[160] Stapledon C.J.M., Tsangari H., Solomon L.B., Campbell D.G., Hurtado P., Krishnan R., Atkins G.J. (2019). Human osteocyte expression of Nerve Growth Factor: The effect of Pentosan Polysulphate Sodium (PPS) and implications for pain associated with knee osteoarthritis. PLoS ONE.

[161] Kumagai K., Shirabe S., Miyata N., Murata M., Yamauchi A., Kataoka Y., Niwa M. (2010). Sodium pentosan polysulfate resulted in cartilage improvement in knee osteoarthritis--an open clinical trial. BMC Clin. Pharmacol..

[162] Smith M.M., Ghosh P., Numata Y., Bansal M.K. (1994). The effects of orally administered calcium pentosan polysulfate on inflammation and cartilage degradation produced in rabbit joints by intraarticular injection of a hyaluronate-polylysine complex. Arthritis Rheum..

[163] Wu J., Shimmon S., Paton S., Daly C., Goldschlager T., Gronthos S., Zannettino A.C.W., Ghosh P. (2017). Pentosan polysulfate binds to STRO-1(+) mesenchymal progenitor cells, is internalized, and modifies gene expression: A novel approach of pre-programing stem cells for therapeutic application requiring their chondrogenesis. Stem Cell Res. Ther..

[164] Daly C.D., Ghosh P., Zannettino A.C.W., Badal T., Shimmon R., Jenkin G., Oehme D., Jain K., Sher I., Vais A. (2018). Mesenchymal progenitor cells primed with pentosan polysulfate promote lumbar intervertebral disc regeneration in an ovine model of microdiscectomy. Spine J..

[165] Ying L., Liang C., Zhang Y., Wang J., Wang C., Xia K., Shi K., Yu C., Yang B., Xu H. (2022). Enhancement of nucleus pulposus repair by glycoengineered adipose-derived mesenchymal cells. Biomaterials.

[166] Kumar P., Kandoi S., Misra R., Vijayalakshmi S., Rajagopal K., Verma R.S. (2019). The mesenchymal stem cell secretome: A new paradigm towards cell-free therapeutic mode in regenerative medicine. Cytokine Growth Factor. Rev..

[167] Cocucci E., Meldolesi J. (2015). Ectosomes and exosomes: Shedding the confusion between extracellular vesicles. Trends Cell Biol..

[168] Korecki C.L., Taboas J.M., Tuan R.S., Iatridis J.C. (2010). Notochordal cell conditioned medium stimulates mesenchymal stem cell differentiation toward a young nucleus pulposus phenotype. Stem Cell Res. Ther..

[169] Sinkemani A., Wang F., Xie Z., Chen L., Zhang C., Wu X. (2020). Nucleus Pulposus Cell Conditioned Medium Promotes Mesenchymal Stem Cell Differentiation into Nucleus Pulposus-Like Cells under Hypoxic Conditions. Stem Cells Int..

[170] Lu K., Li H.Y., Yang K., Wu J.L., Cai X.W., Zhou Y., Li C.Q. (2017). Exosomes as potential alternatives to stem cell therapy for intervertebral disc degeneration: In-vitro study on exosomes in interaction of nucleus pulposus cells and bone marrow mesenchymal stem cells. Stem Cell Res. Ther..

[171] Lan W.R., Pan S., Li H.Y., Sun C., Chang X., Lu K., Jiang C.Q., Zuo R., Zhou Y., Li C.Q. (2019). Inhibition of the Notch1 Pathway Promotes the Effects of Nucleus Pulposus Cell-Derived Exosomes on the Differentiation of Mesenchymal Stem Cells into Nucleus Pulposus-Like Cells in Rats. Stem Cells Int..

[172] Wang H., Tian Y., Wang J., Phillips K.L.E., Binch A.L.A., Dunn S., Cross A., Chiverton N., Zheng Z., Shapiro I.M. (2013). Inflammatory cytokines induce NOTCH signaling in nucleus pulposus cells: Implications in intervertebral disc degeneration. J. Biol. Chem..

[173] Risbud M.V., Shapiro I.M. (2011). Notochordal cells in the adult intervertebral disc: New perspective on an old question. Crit. Rev. Eukaryot. Gene Expr..

[174] Risbud M.V., Schipani E., Shapiro I.M. (2010). Hypoxic regulation of nucleus pulposus cell survival: From niche to notch. Am. J. Pathol..

[175] Hiyama A., Skubutyte R., Markova D., Anderson D.G., Yadla S., Sakai D., Mochida J., Albert T.J., Shapiro I.M., Risbud M.V. (2011). Hypoxia activates the notch signaling pathway in cells of the intervertebral disc: Implications in degenerative disc disease. Arthritis Rheum..

[176] Hirose Y., Johnson Z.I., Schoepflin Z.R., Markova D.Z., Chiba K., Toyama Y., Shapiro I.M., Risbud M.V. (2014). FIH-1-Mint3 axis does not control HIF-1 transcriptional activity in nucleus pulposus cells. J. Biol. Chem..

[177] Vadalà G., Studer R.K., Sowa G., Spiezia F., Iucu C., Denaro V., Gilbertson L.G., Kang J.D. (2008). Coculture of bone marrow mesenchymal stem cells and nucleus pulposus cells modulate gene expression profile without cell fusion. Spine.

[178] Cao C., Zou J., Liu X., Shapiro A., Moral M., Luo Z., Shi Q., Liu J., Yang H., Ebraheim N. (2015). Bone marrow mesenchymal stem cells slow intervertebral disc degeneration through the NF-κB pathway. Spine J..

[179] Li X., Lee J.P., Balian G., Greg Anderson D. (2005). Modulation of chondrocytic properties of fat-derived mesenchymal cells in co-cultures with nucleus pulposus. Connect. Tissue Res..

[180] Chen S., Emery S.E., Pei M. (2009). Coculture of synovium-derived stem cells and nucleus pulposus cells in serum-free defined medium with supplementation of transforming growth factor-beta1: A potential application of tissue-specific stem cells in disc regeneration. Spine.

[181] Potier E., Ito K. (2014). Can notochordal cells promote bone marrow stromal cell potential for nucleus pulposus enrichment? A simplified in vitro system. Tissue Eng. Part A.

[182] Richardson S.M., Walker R.V., Parker S., Rhodes N.P., Hunt J.A., Freemont A.J., Hoyland J.A. (2006). Intervertebral disc cell-mediated mesenchymal stem cell differentiation. Stem Cells.

[183] Wu J., Yu L., Liu Y., Xiao B., Ye X., Zhao H., Xi Y., Shi Z., Wang W. (2023). Hypoxia regulates adipose mesenchymal stem cells proliferation, migration, and nucleus pulposus-like differentiation by regulating endoplasmic reticulum stress via the HIF-1α pathway. J. Orthop. Surg. Res..

[184] Ruan D., Zhang Y., Wang D., Zhang C., Wu J., Wang C., Shi Z., Xin H., Xu C., Li H. (2012). Differentiation of human Wharton’s jelly cells toward nucleus pulposus-like cells after coculture with nucleus pulposus cells in vitro. Tissue Eng. Part A.

[185] Dai J., Wang H., Liu G., Xu Z., Li F., Fang H. (2014). Dynamic compression and co-culture with nucleus pulposus cells promotes proliferation and differentiation of adipose-derived mesenchymal stem cells. J. Biomech..

[186] Allon A.A., Butcher K., Schneider R.A., Lotz J.C. (2012). Structured coculture of mesenchymal stem cells and disc cells enhances differentiation and proliferation. Cells Tissues Organs.

[187] Wei A., Chung S.A., Tao H., Brisby H., Lin Z., Shen B., Ma D.D., Diwan A.D. (2009). Differentiation of rodent bone marrow mesenchymal stem cells into intervertebral disc-like cells following coculture with rat disc tissue. Tissue Eng. Part A.

[188] Cao H., Duan L., Zhang Y., Cao J., Zhang K. (2021). Current hydrogel advances in physicochemical and biological response-driven biomedical application diversity. Signal Transduct. Target. Ther..

[189] Tao Y., Zhou X., Liu D., Li H., Liang C., Li F., Chen Q. (2016). Proportion of collagen type II in the extracellular matrix promotes the differentiation of human adipose-derived mesenchymal stem cells into nucleus pulposus cells. Biofactors.

[190] Sakai D., Mochida J., Yamamoto Y., Nomura T., Okuma M., Nishimura K., Nakai T., Ando K., Hotta T. (2003). Transplantation of mesenchymal stem cells embedded in Atelocollagen gel to the intervertebral disc: A potential therapeutic model for disc degeneration. Biomaterials.

[191] Wang Y., Zhang Y., Chen K., Shao F., Wu Y., Guo C., Wu H., Zhang D., Li W., Kong Q. (2021). Injectable nanostructured colloidal gels resembling native nucleus pulposus as carriers of mesenchymal stem cells for the repair of degenerated intervertebral discs. Mater. Sci. Eng. C Mater. Biol. Appl..

[192] Salzig D., Schmiermund A., Gebauer E., Fuchsbauer H.L., Czermak P. (2011). Influence of porcine intervertebral disc matrix on stem cell differentiation. J. Funct. Biomater..

[193] Vadalà G., Russo F., Musumeci M., D’Este M., Cattani C., Catanzaro G., Tirindelli M.C., Lazzari L., Alini M., Giordano R. (2017). Clinically relevant hydrogel-based on hyaluronic acid and platelet rich plasma as a carrier for mesenchymal stem cells: Rheological and biological characterization. J. Orthop. Res..

[194] Lee K.Y., Mooney D.J. (2012). Alginate: Properties and biomedical applications. Prog. Polym. Sci..

[195] Li Y., Meng H., Liu Y., Lee B.P. (2015). Fibrin gel as an injectable biodegradable scaffold and cell carrier for tissue engineering. Sci. World J..

[196] Smith L.J., Gorth D.J., Showalter B.L., Chiaro J.A., Beattie E.E., Elliott D.M., Mauck R.L., Chen W., Malhotra N.R. (2014). In vitro characterization of a stem-cell-seeded triple-interpenetrating-network hydrogel for functional regeneration of the nucleus pulposus. Tissue Eng. Part A.

[197] Nair M.B., Baranwal G., Vijayan P., Keyan K.S., Jayakumar R. (2015). Composite hydrogel of chitosan-poly(hydroxybutyrate-co-valerate) with chondroitin sulfate nanoparticles for nucleus pulposus tissue engineering. Colloids Surf. B Biointerfaces.

[198] Richardson S.M., Hughes N., Hunt J.A., Freemont A.J., Hoyland J.A. (2008). Human mesenchymal stem cell differentiation to NP-like cells in chitosan-glycerophosphate hydrogels. Biomaterials.

[199] Zareei A., Jiang H., Chittiboyina S., Zhou J., Marin B.P., Lelièvre S.A., Rahimi R. (2020). A lab-on-chip ultrasonic platform for real-time and nondestructive assessment of extracellular matrix stiffness. Lab. Chip.

[200] Wu Y., Jia Z., Liu L., Zhao Y., Li H., Wang C., Tao H., Tang Y., He Q., Ruan D. (2016). Functional Self-Assembled Peptide Nanofibers for Bone Marrow Mesenchymal Stem Cell Encapsulation and Regeneration in Nucleus Pulposus. Artif. Organs.

[201] Russo F., Ambrosio L., Peroglio M., Guo W., Wangler S., Gewiess J., Grad S., Alini M., Papalia R., Vadalà G. (2021). A Hyaluronan and Platelet-Rich Plasma Hydrogel for Mesenchymal Stem Cell Delivery in the Intervertebral Disc: An Organ Culture Study. Int. J. Mol. Sci..

[202] Tsaryk R., Gloria A., Russo T., Anspach L., De Santis R., Ghanaati S., Unger R.E., Ambrosio L., Kirkpatrick C.J. (2015). Collagen-low molecular weight hyaluronic acid semi-interpenetrating network loaded with gelatin microspheres for cell and growth factor delivery for nucleus pulposus regeneration. Acta Biomater..

[203] Calderon L., Collin E., Velasco-Bayon D., Murphy M., O’Halloran D., Pandit A. (2010). Type II collagen-hyaluronan hydrogel--a step towards a scaffold for intervertebral disc tissue engineering. Eur. Cell Mater..

[204] Della Sala F., Biondi M., Guarnieri D., Borzacchiello A., Ambrosio L., Mayol L. (2020). Mechanical behavior of bioactive poly(ethylene glycol) diacrylate matrices for biomedical application. J. Mech. Behav. Biomed. Mater..

[205] Trappmann B., Gautrot J.E., Connelly J.T., Strange D.G., Li Y., Oyen M.L., Cohen Stuart M.A., Boehm H., Li B., Vogel V. (2012). Extracellular-matrix tethering regulates stem-cell fate. Nat. Mater..

[206] Varma D.M., DiNicolas M.S., Nicoll S.B. (2018). Injectable, redox-polymerized carboxymethylcellulose hydrogels promote nucleus pulposus-like extracellular matrix elaboration by human MSCs in a cell density-dependent manner. J. Biomater. Appl..

[207] Lin H.A., Gupta M.S., Varma D.M., Gilchrist M.L., Nicoll S.B. (2016). Lower crosslinking density enhances functional nucleus pulposus-like matrix elaboration by human mesenchymal stem cells in carboxymethylcellulose hydrogels. J. Biomed. Mater. Res. A.

[208] Thorpe A.A., Boyes V.L., Sammon C., Le Maitre C.L. (2016). Thermally triggered injectable hydrogel, which induces mesenchymal stem cell differentiation to nucleus pulposus cells: Potential for regeneration of the intervertebral disc. Acta Biomater..

[209] Huang X., Chen D., Liang C., Shi K., Zhou X., Zhang Y., Li Y., Chen J., Xia K., Shu J. (2022). Swelling-Mediated Mechanical Stimulation Regulates Differentiation of Adipose-Derived Mesenchymal Stem Cells for Intervertebral Disc Repair Using Injectable UCST Microgels. Adv. Healthc. Mater..

[210] Liang C., Li H., Li C., Yang Z., Zhou X., Tao Y., Xiao Y., Li F., Chen Q. (2012). Fabrication of a Layered Microstructured Polymeric Microspheres as a Cell Carrier for Nucleus Pulposus Regeneration. J. Biomater. Sci. Polym. Ed..

[211] Liang C.Z., Li H., Tao Y.Q., Zhou X.P., Yang Z.R., Xiao Y.X., Li F.C., Han B., Chen Q.X. (2012). Dual delivery for stem cell differentiation using dexamethasone and bFGF in/on polymeric microspheres as a cell carrier for nucleus pulposus regeneration. J. Mater. Sci. Mater. Med..

[212] Frith J.E., Cameron A.R., Menzies D.J., Ghosh P., Whitehead D.L., Gronthos S., Zannettino A.C., Cooper-White J.J. (2013). An injectable hydrogel incorporating mesenchymal precursor cells and pentosan polysulphate for intervertebral disc regeneration. Biomaterials.

[213] Frith J.E., Menzies D.J., Cameron A.R., Ghosh P., Whitehead D.L., Gronthos S., Zannettino A.C., Cooper-White J.J. (2014). Effects of bound versus soluble pentosan polysulphate in PEG/HA-based hydrogels tailored for intervertebral disc regeneration. Biomaterials.

[214] Vickers L., Thorpe A.A., Snuggs J., Sammon C., Le Maitre C.L. (2019). Mesenchymal stem cell therapies for intervertebral disc degeneration: Consideration of the degenerate niche. JOR Spine.

[215] Gan Y., Li S., Li P., Xu Y., Wang L., Zhao C., Ouyang B., Tu B., Zhang C., Luo L. (2016). A Controlled Release Codelivery System of MSCs Encapsulated in Dextran/Gelatin Hydrogel with TGF-β3-Loaded Nanoparticles for Nucleus Pulposus Regeneration. Stem Cells Int..

[216] Mwale F., Girard-Lauriault P.L., Wang H.T., Lerouge S., Antoniou J., Wertheimer M.R. (2006). Suppression of genes related to hypertrophy and osteogenesis in committed human mesenchymal stem cells cultured on novel nitrogen-rich plasma polymer coatings. Tissue Eng..

[217] Hansson A., Wenger A., Henriksson H.B., Li S., Johansson B.R., Brisby H. (2017). The direction of human mesenchymal stem cells into the chondrogenic lineage is influenced by the features of hydrogel carriers. Tissue Cell.

[218] Purmessur D., Schek R.M., Abbott R.D., Ballif B.A., Godburn K.E., Iatridis J.C. (2011). Notochordal conditioned media from tissue increases proteoglycan accumulation and promotes a healthy nucleus pulposus phenotype in human mesenchymal stem cells. Arthritis Res. Ther..

[219] Wang J., Tao Y., Zhou X., Li H., Liang C., Li F., Chen Q.X. (2016). The potential of chondrogenic pre-differentiation of adipose-derived mesenchymal stem cells for regeneration in harsh nucleus pulposus microenvironment. Exp. Biol. Med..

[220] Wang H., Zhou Y., Huang B., Liu L.T., Liu M.H., Wang J., Li C.Q., Zhang Z.F., Chu T.W., Xiong C.J. (2014). Utilization of stem cells in alginate for nucleus pulposus tissue engineering. Tissue Eng. Part A.

[221] Tsujimoto T., Sudo H., Todoh M., Yamada K., Iwasaki K., Ohnishi T., Hirohama N., Nonoyama T., Ukeba D., Ura K. (2018). An acellular bioresorbable ultra-purified alginate gel promotes intervertebral disc repair: A preclinical proof-of-concept study. EBioMedicine.

[222] Li Y.Y., Diao H.J., Chik T.K., Chow C.T., An X.M., Leung V., Cheung K.M., Chan B.P. (2014). Delivering mesenchymal stem cells in collagen microsphere carriers to rabbit degenerative disc: Reduced risk of osteophyte formation. Tissue Eng. Part A.

[223] Bertolo A., Häfner S., Taddei A.R., Baur M., Pötzel T., Steffen F., Stoyanov J. (2015). Injectable microcarriers as human mesenchymal stem cell support and their application for cartilage and degenerated intervertebral disc repair. Eur. Cell Mater..

[224] Zeng Y., Feng S., Liu W., Fu Q., Li Y., Li X., Chen C., Huang C., Ge Z., Du Y. (2017). Preconditioning of mesenchymal stromal cells toward nucleus pulposus-like cells by microcryogels-based 3D cell culture and syringe-based pressure loading system. J. Biomed. Mater. Res. B Appl. Biomater..

[225] Bian Z., Sun J. (2015). Development of a KLD-12 polypeptide/TGF-β1-tissue scaffold promoting the differentiation of mesenchymal stem cell into nucleus pulposus-like cells for treatment of intervertebral disc degeneration. Int. J. Clin. Exp. Pathol..

[226] Wang Z., Yang H., Xu X., Hu H., Bai Y., Hai J., Cheng L., Zhu R. (2023). Ion elemental-optimized layered double hydroxide nanoparticles promote chondrogenic differentiation and intervertebral disc regeneration of mesenchymal stem cells through focal adhesion signaling pathway. Bioact. Mater..

[227] Bertolo A., Mehr M., Aebli N., Baur M., Ferguson S.J., Stoyanov J.V. (2012). Influence of different commercial scaffolds on the in vitro differentiation of human mesenchymal stem cells to nucleus pulposus-like cells. Eur. Spine J..

[228] Naqvi S.M., Buckley C.T. (2015). Differential response of encapsulated nucleus pulposus and bone marrow stem cells in isolation and coculture in alginate and chitosan hydrogels. Tissue Eng. Part A.

[229] Xu J., Liu S., Wang S., Qiu P., Chen P., Lin X., Fang X. (2019). Decellularised nucleus pulposus as a potential biologic scaffold for disc tissue engineering. Mater. Sci. Eng. C Mater. Biol. Appl..

[230] Yuan M., Yeung C.W., Li Y.Y., Diao H., Cheung K.M.C., Chan D., Cheah K., Chan P.B. (2013). Effects of nucleus pulposus cell-derived acellular matrix on the differentiation of mesenchymal stem cells. Biomaterials.

[231] Yu L., Liu Y., Wu J., Wang S., Yu J., Wang W., Ye X. (2021). Genipin Cross-Linked Decellularized Nucleus Pulposus Hydrogel-Like Cell Delivery System Induces Differentiation of ADSCs and Retards Intervertebral Disc Degeneration. Front. Bioeng. Biotechnol..

[232] Peng Y., Huang D., Li J., Liu S., Qing X., Shao Z. (2020). Genipin-crosslinked decellularized annulus fibrosus hydrogels induces tissue-specific differentiation of bone mesenchymal stem cells and intervertebral disc regeneration. J. Tissue Eng. Regen. Med..

[233] Lin X., Fang X., Wang Q., Hu Z., Chen K., Shan Z., Chen S., Wang J., Mo J., Ma J. (2016). Decellularized allogeneic intervertebral disc: Natural biomaterials for regenerating disc degeneration. Oncotarget.

[234] Frauchiger D.A., Heeb S.R., May R.D., Wöltje M., Benneker L.M., Gantenbein B. (2018). Differentiation of MSC and annulus fibrosus cells on genetically engineered silk fleece-membrane-composites enriched for GDF-6 or TGF-β3. J. Orthop. Res..

[235] Bhunia B.K., Kaplan D.L., Mandal B.B. (2018). Silk-based multilayered angle-ply annulus fibrosus construct to recapitulate form and function of the intervertebral disc. Proc. Natl. Acad. Sci. USA.

[236] Merceron C., Mangiavini L., Robling A., Wilson T.L., Giaccia A.J., Shapiro I.M., Schipani E., Risbud M.V. (2014). Loss of HIF-1alpha in the notochord results in cell death and complete disappearance of the nucleus pulposus. PLoS ONE.

[237] Mylonis I., Simos G., Paraskeva E. (2019). Hypoxia-Inducible Factors and the Regulation of Lipid Metabolism. Cells.

[238] Fujita N., Chiba K., Shapiro I.M., Risbud M.V. (2012). HIF-1α and HIF-2α degradation is differentially regulated in nucleus pulposus cells of the intervertebral disc. J. Bone Miner. Res..

[239] Silagi E.S., Schipani E., Shapiro I.M., Risbud M.V. (2021). The role of HIF proteins in maintaining the metabolic health of the intervertebral disc. Nat. Rev. Rheumatol..

[240] Silagi E.S., Schoepflin Z.R., Seifert E.L., Merceron C., Schipani E., Shapiro I.M., Risbud M.V. (2018). Bicarbonate Recycling by HIF-1-Dependent Carbonic Anhydrase Isoforms 9 and 12 Is Critical in Maintaining Intracellular pH and Viability of Nucleus Pulposus Cells. J. Bone Miner. Res..

[241] Fujita N., Hirose Y., Tran C.M., Chiba K., Miyamoto T., Toyama Y., Shapiro I.M., Risbud M.V. (2014). HIF-1-PHD2 axis controls expression of syndecan 4 in nucleus pulposus cells. FASEB J..

[242] Müller J., Benz K., Ahlers M., Gaissmaier C., Mollenhauer J. (2011). Hypoxic conditions during expansion culture prime human mesenchymal stromal precursor cells for chondrogenic differentiation in three-dimensional cultures. Cell Transplant..

[243] Peck S.H., Bendigo J.R., Tobias J.W., Dodge G.R., Malhotra N.R., Mauck R.L., Smith L.J. (2021). Hypoxic Preconditioning Enhances Bone Marrow-Derived Mesenchymal Stem Cell Survival in a Low Oxygen and Nutrient-Limited 3D Microenvironment. Cartilage.

[244] Li H., Tao Y., Liang C., Han B., Li F., Chen G., Chen Q. (2013). Influence of hypoxia in the intervertebral disc on the biological behaviors of rat adipose- and nucleus pulposus-derived mesenchymal stem cells. Cells Tissues Organs.

[245] Choi H., Chaiyamongkol W., Doolittle A.C., Johnson Z.I., Gogate S.S., Schoepflin Z.R., Shapiro I.M., Risbud M.V. (2018). COX-2 expression mediated by calcium-TonEBP signaling axis under hyperosmotic conditions serves osmoprotective function in nucleus pulposus cells. J. Biol. Chem..

[246] Favale N.O., Casali C.I., Lepera L.G., Pescio L.G., Fernández-Tome M.C. (2009). Hypertonic induction of COX2 expression requires TonEBP/NFAT5 in renal epithelial cells. Biochem. Biophys. Res. Commun..

[247] Johnson Z.I., Shapiro I.M., Risbud M.V. (2014). Extracellular osmolarity regulates matrix homeostasis in the intervertebral disc and articular cartilage: Evolving role of TonEBP. Matrix Biol..

[248] Hiyama A., Gajghate S., Sakai D., Mochida J., Shapiro I.M., Risbud M.V. (2009). Activation of TonEBP by calcium controls {beta}1,3-glucuronosyltransferase-I expression, a key regulator of glycosaminoglycan synthesis in cells of the intervertebral disc. J. Biol. Chem..

[249] Hiyama A., Gogate S.S., Gajghate S., Mochida J., Shapiro I.M., Risbud M.V. (2010). BMP-2 and TGF-beta stimulate expression of beta1,3-glucuronosyl transferase 1 (GlcAT-1) in nucleus pulposus cells through AP1, TonEBP, and Sp1: Role of MAPKs. J. Bone Miner. Res..

[250] Gogate S.S., Fujita N., Skubutyte R., Shapiro I.M., Risbud M.V. (2012). Tonicity enhancer binding protein (TonEBP) and hypoxia-inducible factor (HIF) coordinate heat shock protein 70 (Hsp70) expression in hypoxic nucleus pulposus cells: Role of Hsp70 in HIF-1α degradation. J. Bone Miner. Res..

[251] Zhang Y., Wang Y., Zhou X., Wang J., Shi M., Wang J., Li F., Chen Q. (2020). Osmolarity controls the differentiation of adipose-derived stem cells into nucleus pulposus cells via histone demethylase KDM4B. Mol. Cell Biochem..

[252] Caron M.M., van der Windt A.E., Emans P.J., van Rhijn L.W., Jahr H., Welting T.J. (2013). Osmolarity determines the in vitro chondrogenic differentiation capacity of progenitor cells via nuclear factor of activated T-cells 5. Bone.

[253] Li H., Wang J., Li F., Chen G., Chen Q. (2018). The Influence of Hyperosmolarity in the Intervertebral Disc on the Proliferation and Chondrogenic Differentiation of Nucleus Pulposus-Derived Mesenchymal Stem Cells. Cells Tissues Organs.

[254] Potočar U., Hudoklin S., Kreft M.E., Završnik J., Božikov K., Fröhlich M. (2016). Adipose-Derived Stem Cells Respond to Increased Osmolarities. PLoS ONE.

[255] Ahmadyan S., Kabiri M., Hanaee-Ahvaz H., Farazmand A. (2018). Osmolyte Type and the Osmolarity Level Affect Chondrogenesis of Mesenchymal Stem Cells. Appl. Biochem. Biotechnol..

[256] Angele P., Schumann D., Angele M., Kinner B., Englert C., Hente R., Füchtmeier B., Nerlich M., Neumann C., Kujat R. (2004). Cyclic, mechanical compression enhances chondrogenesis of mesenchymal progenitor cells in tissue engineering scaffolds. Biorheology.

[257] Huang C.Y., Hagar K.L., Frost L.E., Sun Y., Cheung H.S. (2004). Effects of cyclic compressive loading on chondrogenesis of rabbit bone-marrow derived mesenchymal stem cells. Stem Cells.

[258] Huang C.Y., Reuben P.M., Cheung H.S. (2005). Temporal expression patterns and corresponding protein inductions of early responsive genes in rabbit bone marrow-derived mesenchymal stem cells under cyclic compressive loading. Stem Cells.

[259] Gan Y., Tu B., Li P., Ye J., Zhao C., Luo L., Zhang C., Zhang Z., Zhu L., Zhou Q. (2018). Low Magnitude of Compression Enhances Biosynthesis of Mesenchymal Stem Cells towards Nucleus Pulposus Cells via the TRPV4-Dependent Pathway. Stem Cells Int..

[260] Li Z., Kupcsik L., Yao S.J., Alini M., Stoddart M.J. (2010). Mechanical load modulates chondrogenesis of human mesenchymal stem cells through the TGF-beta pathway. J. Cell Mol. Med..

[261] Mouw J.K., Connelly J.T., Wilson C.G., Michael K.E., Levenston M.E. (2007). Dynamic compression regulates the expression and synthesis of chondrocyte-specific matrix molecules in bone marrow stromal cells. Stem Cells.

[262] Zhang Y., Tang C.L., Chen W.J., Zhang Q., Wang S.L. (2015). Dynamic compression combined with exogenous SOX-9 promotes chondrogenesis of adipose-derived mesenchymal stem cells in PLGA scaffold. Eur. Rev. Med. Pharmacol. Sci..

[263] Li Z., Yao S.J., Alini M., Stoddart M.J. (2010). Chondrogenesis of human bone marrow mesenchymal stem cells in fibrin-polyurethane composites is modulated by frequency and amplitude of dynamic compression and shear stress. Tissue Eng. Part A.

[264] Liang H., Chen S., Huang D., Deng X., Ma K., Shao Z. (2018). Effect of Compression Loading on Human Nucleus Pulposus-Derived Mesenchymal Stem Cells. Stem Cells Int..

[265] Liang N.E., Griffin M.F., Berry C.E., Parker J.B., Downer M.A., Wan D.C., Longaker M.T. (2023). Attenuating Chronic Fibrosis: Decreasing Foreign Body Response with Acellular Dermal Matrix. Tissue Eng. Part B Rev..

[266] Peng Y., Qing X., Shu H., Tian S., Yang W., Chen S., Lin H., Lv X., Zhao L., Chen X. (2021). Proper animal experimental designs for preclinical research of biomaterials for intervertebral disc regeneration. Biomater. Transl..

[267] Silagi E.S., Novais E.J., Bisetto S., Telonis A.G., Snuggs J., Le Maitre C.L., Qiu Y., Kurland I.J., Shapiro I.M., Philp N.J. (2020). Lactate Efflux From Intervertebral Disc Cells Is Required for Maintenance of Spine Health. J. Bone Miner. Res..

[268] Sudo H., Minami A. (2010). Regulation of apoptosis in nucleus pulposus cells by optimized exogenous Bcl-2 overexpression. J. Orthop. Res..

[269] Hiyama A., Mochida J., Iwashina T., Omi H., Watanabe T., Serigano K., Tamura F., Sakai D. (2008). Transplantation of mesenchymal stem cells in a canine disc degeneration model. J. Orthop. Res..

[270] Kaneyama S., Nishida K., Takada T., Suzuki T., Shimomura T., Maeno K., Kurosaka M., Doita M. (2008). Fas ligand expression on human nucleus pulposus cells decreases with disc degeneration processes. J. Orthop. Sci..

[271] García-Sancho J., Sánchez A., Vega A., Noriega D.C., Nocito M. (2017). Influence of HLA Matching on the Efficacy of Allogeneic Mesenchymal Stromal Cell Therapies for Osteoarthritis and Degenerative Disc Disease. Transplant. Direct.

[272] Soufi K.H., Castillo J.A., Rogdriguez F.Y., DeMesa C.J., Ebinu J.O. (2023). Potential Role for Stem Cell Regenerative Therapy as a Treatment for Degenerative Disc Disease and Low Back Pain: A Systematic Review. Int. J. Mol. Sci..

